# Dynamics of a Dual SARS-CoV-2 Lineage Co-Infection on a Prolonged Viral Shedding COVID-19 Case: Insights into Clinical Severity and Disease Duration

**DOI:** 10.3390/microorganisms9020300

**Published:** 2021-02-02

**Authors:** Nicole Pedro, Cláudio N. Silva, Ana C. Magalhães, Bruno Cavadas, Ana M. Rocha, Ana C. Moreira, Maria Salomé Gomes, Diogo Silva, Joana Sobrinho-Simões, Angélica Ramos, Maria J. Cardoso, Rita Filipe, Pedro Palma, Filipa Ceia, Susana Silva, João T. Guimarães, António Sarmento, Verónica Fernandes, Luisa Pereira, Margarida Tavares

**Affiliations:** 1 i3S—Instituto de Investigação e Inovação em Saúde, Universidade do Porto, 4200-135 Porto, Portugal; npedro@ipatimup.pt (N.P.); acmagalhaes@ipatimup.pt (A.C.M.); bcavadas@ipatimup.pt (B.C.); anar@ipatimup.pt (A.M.R.); ana.s.moreira@ibmc.up.pt (A.C.M.); sgomes@ibmc.up.pt (M.S.G.); antonio.sarmento@chsj.min-saude.pt (A.S.); vfernandes@ipatimup.pt (V.F.); 2 Ipatimup—Instituto de Patologia e Imunologia Molecular, Universidade do Porto, 4200-135 Porto, Portugal; 3 ICBAS—Instituto de Ciências Biomédicas Abel Salazar, Universidade do Porto, 4050-313 Porto, Portugal; 4 CHUSJ—Centro Hospitalar Universitário S. João, 4200-319 Porto, Portugal; claudio.j.n.silva@gmail.com (C.N.S.); diogomagalhaes444@gmail.com (D.S.); jssimoes@chsj.min-saude.pt (J.S.-S.); angelicacostaramos@gmail.com (A.R.); princesamjc@gmail.com (M.J.C.); rita.filipe.02@gmail.com (R.F.); pedropalmamartins@gmail.com (P.P.); fsfceia@gmail.com (F.C.); susanamaiasilva@gmail.com (S.S.); jtguimar@med.up.pt (J.T.G.); margarida.tavares@chsj.min-saude.pt (M.T.); 5 FMUP—Faculdade de Medicina, Universidade do Porto, 4200-319 Porto, Portugal; 6 IBMC—Instituto de Biologia Molecular e Celular, Universidade do Porto, 4200-135 Porto, Portugal; 7 EPIUnit—Instituto de Saúde Pública, Universidade do Porto, 4050-091 Porto, Portugal

**Keywords:** COVID-19 and SARS-CoV-2, viral shedding, co-infection, viral whole-genome sequencing, polygenic risk score

## Abstract

A few molecularly proven severe acute respiratory syndrome coronavirus 2 (SARS-CoV-2) cases of symptomatic reinfection are currently known worldwide, with a resolved first infection followed by a second infection after a 48 to 142-day intervening period. We report a multiple-component study of a clinically severe and prolonged viral shedding coronavirus disease 2019 (COVID-19) case in a 17-year-old Portuguese female. She had two hospitalizations, a total of 19 RT-PCR tests, mostly positive, and criteria for releasing from home isolation at the end of 97 days. The viral genome was sequenced in seven serial samples and in the diagnostic sample from her infected mother. A human genome-wide array (>900 K) was screened on the seven samples, and in vitro culture was conducted on isolates from three late samples. The patient had co-infection by two SARS-CoV-2 lineages, which were affiliated in distinct clades and diverging by six variants. The 20A lineage was absolute at the diagnosis (shared with the patient’s mother), but nine days later, the 20B lineage had 3% frequency, and two months later, the 20B lineage had 100% frequency. The 900 K profiles confirmed the identity of the patient in the serial samples, and they allowed us to infer that she had polygenic risk scores for hospitalization and severe respiratory disease within the normal distributions for a Portuguese population cohort. The early-on dynamic co-infection may have contributed to the severity of COVID-19 in this otherwise healthy young patient, and to her prolonged SARS-CoV-2 shedding profile.

## 1. Introduction

The new coronavirus disease 2019 (COVID-19), caused by the severe acute respiratory syndrome coronavirus 2 (SARS-CoV-2), was identified in Wuhan, China [1] and rapidly disseminated at a global scale. Whether or not SARS-CoV-2 infection induces long-term protective immunity remains an open question, and we have to wait several months before knowing if current vaccines allow establishing herd immunity [2]. Closely related with the immunity issue is the puzzling observation of persistent viral shedding detectable in real-time reverse transcriptase–polymerase chain reaction (RT-PCR) tests, even after symptom resolution [3]. Usually, SARS-CoV-2 viral load peaks in the first week of illness, compared with 10–14 days for SARS-CoV and 7–10 days for MERS-CoV. However, long shedding cases are not rare, and a survey of 378 Covid-19 patients from Wuhan revealed that 36 continued to shed the virus longer than 30 days (mean of 54 days; longest 83 days; [4]). This extended or recurrent positivity could be due to (1) a reactivation of the virus after a period of clinical latency; (2) SARS-CoV-2 reinfection; (3) or simply RT-PCR tests detecting viral remains and not necessarily active viral particles. To untangle between the two first possibilities, it is necessary to perform viral whole-genome sequencing and detect genetically distinct lineages of SARS-CoV-2 in each of the disease episodes; of course, reinfections by the same lineage may remain unnoticed [5]. To check for the last possibility, the inclusion of infectivity studies might help understand if the virus retains both viability and integrity [6]. It is becoming evident that for most SARS-CoV-2 infection cases, in common with the related SARS-CoV and MERS-CoV viruses, the duration of viable viruses is short, despite long shedding [7].

At the moment, a few molecularly proven SARS-CoV-2 symptomatic reinfections have been published worldwide [8], namely in Hong-Kong [9], Belgium [10], Ecuador [11], and the USA [12]. After being considered recovered, these four patients presented a second infection by a genetically distinct SARS-CoV-2 lineage after a 48 to 142-day intervening period. The latter two patients displayed a more severe disease in the second episode, which is in accordance to the hypothesis of an antibody-dependent enhancement (ADE) to SARS-CoV-2 [13]. The number of reinfection cases must be higher than currently reported, as they are easily missed when asymptomatic. So far, two asymptomatic reinfections were detected in Indian healthcare workers, whom were routinely screened in their workplace [14]. Although these two cases were asymptomatic in the first and second infections, the viral load was higher in the second one, as inferred from the RT-PCR cycle threshold. Another theoretical possibility is the occurrence of reinfection while the first infection was not yet cured, which is a situation best described as co-infection. This hypothesis was advanced by the authors for the USA reinfection case [12], but it has not been thoroughly studied.

In this work, we report a molecularly proven dynamic early-on co-infection by two genetically distinct SARS-CoV-2 lineages. This was a prolonged viral shedding case (97 days long), with a first severe disease manifestation, followed by a short-second hospitalization episode, in an otherwise healthy young female.

## 2. Materials and Methods

### 2.1. RT-PCR and Antibody Testing

For the detection of viral RNA by RT-PCR, each tube sample contained a nasopharyngeal and an oropharyngeal swab immersed in virus preservation solution. RNA was extracted with the Qiacube extractor by using the spin-column Qiamp virus minikit (Qiagen, Hilden, Germany). The reported RT-PCR results for the SARS-CoV-2 E gene were obtained with the LightCycler^®^ Multiplex RNA Virus Master (Roche Life Science, Penzberg, Germany) at a LightCycler^®^ 480 Instrument II (Roche Life Science, Penzberg, Germany), and it included a RNA extraction control (LightMix^®^ Modular EAV RNA Extraction Control; Tib Molbiol, Berlin, Germany). Positive and negative controls were routinely included with each batch of tests. Relative quantification of the sample crossing points (cp) was automatically inferred with the LightCycler software.

The serological test used was a chemiluminescent microparticle immunoassay for the qualitative detection of IgG against SARS-CoV-2 nucleoprotein (Abbott Diagnostics, Chicago, IL, USA). Serum samples were run on the Abbott Architect instrument following the manufacturer’s instructions. The amount of IgG antibodies to SARS-CoV-2 in each sample was determined by comparing its chemiluminescent relative light unit (RLU) to the calibrator RLU (index S/C). A signal/cut-off (S/CO) ratio of ≥1.4 was interpreted as reactive and an S/CO ratio of <1.4 was interpreted as non-reactive.

### 2.2. Viral Whole Genome-Sequencing and Phylogenetic Analysis

Seven serial extracted viral RNA samples from the patient and the diagnosis sample from her mother were also used for the viral whole-genome sequencing. The protocol consisted of reverse-transcription with the SuperScriptTM VILOTM cDNA synthesis Kit (Thermo Fisher Scientific, Waltham, MA, USA); PCR enrichment of the SARS-CoV-2 genome, and five human gene expression controls with the Ion AmpliSeqTM SARS-CoV-2 Research Panel; library construction with the Ion AmpliSeqTM Library Kit Plus; library quantification and size range verification at the 2200 TapeStation Automated Electrophoresis System, using the High Sensitivity DNA ScreenTape (Agilent Technologies, Santa Clara, CA, USA); next-generation sequencing (NGS) on the Ion S5XL system with the Ion 530™ chip; raw data extracted with the Ion Torrent pipeline. The bioinformatic pipeline consisted of alignment of the raw data versus the reference genome (accession number NC_045512.2) with the BWA tool; variant calling with three tools, FreeBayes, BCFtools, and GATK, with the editing of variants identified in at least two; variant annotation with SnpEff; consensus sequence was inferred with Bcftools [15]. Phylogenetic analysis and lineage/clade affiliation were done by merging the consensus sequences with publically available SARS-CoV-2 whole genomes from ViPR [16] with Mafft [17] (first 130bp and last 50bp were masked). The rooted phylogenetic tree was obtained with IQ-TREE 2 [18] and visualized using Interactive Tree Of Live version 4 [19].

### 2.3. Genome-Wide Array Screening and Calculation of the Polygenic Risk Score

The genotyping of over 900,000 SNPs (900 K) was attained with the Axiom™ Precision Medicine Diversity Array and the GeneTitan Multi-Channel (MC) Instrument (Thermo Fisher Scientific, Waltham, MA, USA). The laboratorial procedure was adapted to allow the use of samples extracted from the nasopharyngeal swabs by increasing the time of the whole genome amplification step from 24 to 48h. Genotyping was inferred with the Array Power Tool, and the PADRE algorithm [20] was used for the accurate estimation of shared ancestry (in this case, identity). These data were also used to calculate the polygenic risk score (PRS) in the Portuguese population cohort (*n* = 198) to contextualize the patient score. The PMDA variants were uploaded into the Michigan Imputation Server (https://imputationserver.sph.umich.edu/index.html#!) and imputed based on the Haplotype Reference Consortium panel. The PRS values were calculated from the significant odd ratios (*p*-value < 10^−5^) reported online by the COVID-19 host genetics initiative (https://www.covid19hg.org/) [21], for two phenotype cohorts from data released in 30th September 2020 (COVID19-hg GWAS meta-analyses round 4 (alpha)): A2_ALL dataset of “very severe respiratory confirmed covid” (*n* = 2972) vs. population (*n* = 284,472); and B2_ALL dataset of “hospitalised covid” (*n* = 6492) vs. population (*n* = 1,012,809). Linked variants were removed in plink using the flags—clump-r2 0.4—clump-kb 250; we ended up with 91 variants for the first dataset and 79 for the second. Plink was also used to estimate PRS values via an additive model, as the sum of the risk alleles, weighted by the effect size estimates from the genome-wide association study.

### 2.4. In Vitro Culture of the Virus

To ascertain if the virus was still viable in the samples after the second hospitalization, in vitro culture in Vero cells (ATCC CCL-81; ATCC, Manassas, VA, USA) was performed, followed by SARS-CoV-2 spike antibody (GeneTex, Irvine, CA, USA) immunofluorescence detection. Images were acquired on the IN Cell Analyzer 2000 (Cytiva, Marlborough, MA, USA). Following a first inoculation of the samples for 96 h, the resulting supernatant was transferred to 96 wells and inoculated for 24 h, 48 h, and 72 h in duplicates to assess residual virus particles not detected in the first culture. We included a recently diagnosed sample from another patient to guarantee the assay was able to detect the viral particles.

## 3. Results

### 3.1. Clinical Features

A previously healthy 17-year-old female presented to the local hospital emergency department in March 8th reporting a 9-day history of sustained fever, dry cough, pleuritic chest pain, and vomiting. She was hemodynamically stable (105/63 mmHg blood pressure), but tachypnoeic (28 cpm respiratory rate), hypoxic to 88% on room air, and febrile to 101.3 °F. Her chest computed tomography (CT) scan revealed extensive bilateral subpleural ground-glass opacities (GGO) with areas of air-space consolidation, and she was admitted for etiological investigation and treatment (Figure S1). A nasopharyngeal swab performed during the initial workup detected SARS-CoV-2 RNA (Table S2), and the patient was transferred to our referral center for Emerging Infectious Diseases at Centro Hospitalar Universitário de São João (CHUSJ). At admission lymphopenia, a mild increased level of C-reactive protein and normal prothrombin and activated partial thromboplastin times were seen (Table S1). Due to worsening respiratory status and increasing supplementary oxygen demands, she was placed on High-Flow Nasal Oxygen (HFNO). After a 12-h HFNO trial without improvement, she was admitted to our Infectious Diseases ICU on the 12th day of symptoms, beginning an off-label 5-day hydroxychloroquine course.

After six days of inpatient care, she complained of left upper limb pain with signs consistent with deep vein thrombosis associated with indwelling peripheral venous catheter. She also complained of worsening bilateral pleuritic chest pain. Accordingly, D-dimer levels increased to 2.47 μg/mL and a chest angio-CT scan showed lower left lung lobe peripheral infarction (Figure S2). Anticoagulation with low-molecular-weight-heparin was started, the supplementary oxygen demands gradually decreased, and she was discharged home 9 days after admission completely asymptomatic with an oxygen saturation of 99% on room air. RT-PCR tests of nasopharyngeal specimens remained positive for SARS-CoV-2 RNA at discharge. The patient was asked to continue isolating at home until attaining two consecutive negative tests.

In mid-May, nearly two months after discharge, she was re-admitted with headaches, fever, myalgia, and right pleuritic chest pain. Her vital signs were 110/52 mmHg blood pressure, 112 bpm heart rate, and 98.7 °F body temperature. Her peripheral blood oxygen saturation was 97% on room air, and all of the blood work values were within the normal range, non-immunocompromised (Tables S1 and S3). After an extensive microbiologic workup (Table S2), other infectious etiologies were ruled out. The chest CT scan (Figure S3) revealed improved aeration of the lungs and resolving GGO features. Repeated SARS-CoV-2 RT-PCR was still positive.

The symptoms resolved in 2 days. Anti-SARS-CoV-2 IgG antibodies were detected and reactive by immunoassay on May 26th (relative light unit (RLU) index sample/calibrator (S/C) of 7.16), on 9th June (S/C index of 6.89) and again on October 22nd, almost 8 months after first symptoms (S/C index of 2.26).

The 41-year-old patient’s mother was the only household contact who developed symptomatic COVID-19. In this case, the symptoms were mild: limited to a one-day low-grade fever, and scarce dry cough starting 10 days after the daughter’s symptom onset.

### 3.2. Viral Viability and Genome Analysis

**Figure 1:**
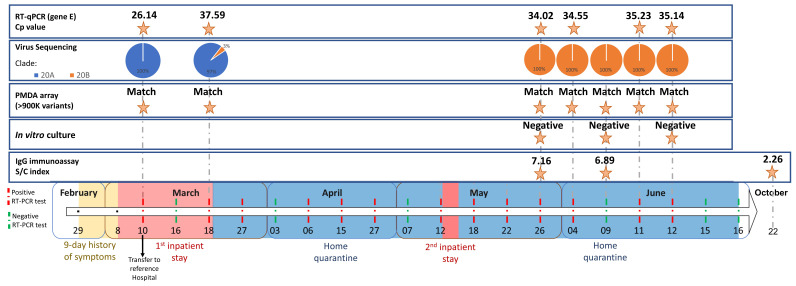
Timeline of the reported coronavirus disease 2019 (COVID-19) case and main results of the multi-component study conducted on the serial samples. The timeline bar indicates the periods of: symptom onset (yellow); inpatient stays (pink; the black arrow indicates the moment when the patient was transferred to the referral center for Emerging Infectious Diseases at Centro Hospitalar Universitário de São João—CHUSJ); and, home quarantine (blue). It also includes the dates of all the 19 RT-PCR tests performed (green for negative result and red for positive result). The molecular tests were focused either on the virus (RT-PCR crossing point, proportion of clade/lineage affiliation inferred from viral whole-genome sequencing, and in vitro culture) or the host (array containing > 900 K variants and IgG antibody immunoassay reported as sample/calibrator relative light unit index).

**Figure 2:**
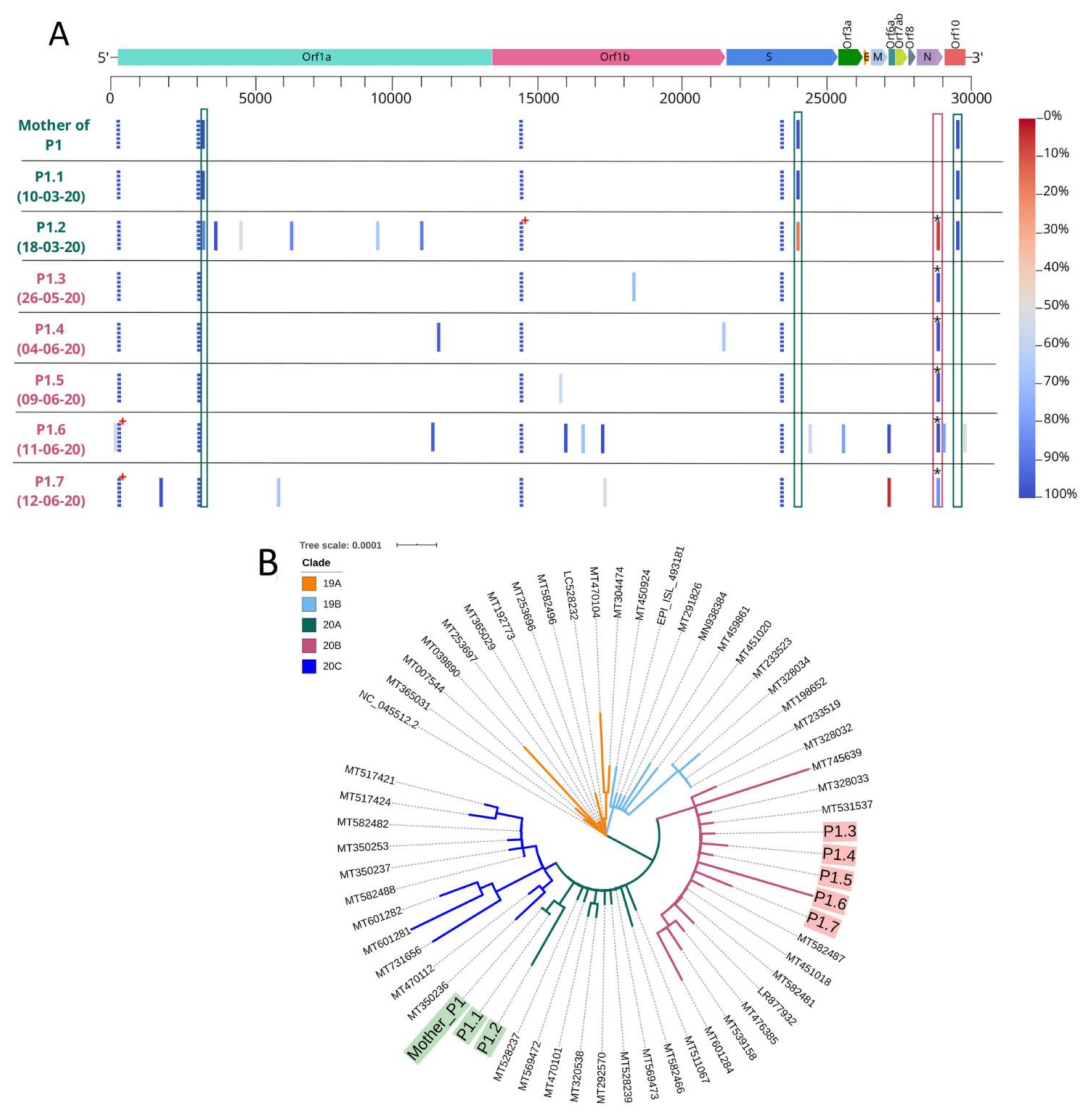
Detailed sequence diversity of the severe acute respiratory syndrome coronavirus 2 (SARS-CoV-2) isolates from the various samples. (**A**)—Representation of the SARS-CoV-2 genome (genes are indicated) and the variants (only those with 50% frequency in at least one of the eight samples) sequenced in the isolates from the patient serial samples (P1.1 to P1.7) and her mother’s diagnostic sample. Date of sample collections are indicated below the patient samples ID, and the color code reflects the main 20A (in green) or 20B (in pink) SARS-CoV-2 lineage. The gradient bar indicates the frequency of the variants. For easier visualization, the shared variants between 20A and 20B lineages are indicated in doted bars. The boxes highlight either the specific 20A variants (in green) or the 20B-defining variants (in pink; the asterisk calls the attention to the three sequential variants, G28881A-G28882A-G28883C). The red cross indicates missing positions, matching known regions of the SARS-CoV-2 genome that are difficult to sequence. (**B**)—Phylogenetic tree of the main SARS-CoV-2 clades known so far (19A, 19B, 20A, 20B, and 20C) and the sequences reported here (following the color scheme of **A**).

The sequencing of the viral genome in the serial samples from the patient revealed very interesting results (Figure 1 and Figure 2). At the diagnosis (sample P1.1), she displayed 100% of a 20A affiliated haplotype, bearing the basal C241T-C3037T-C14408T-A23403G variants (in relation to the reference sequence), and additional C3140T-G24077T-C295557 variants. The G24077T variant has been extensively observed in Lombardy, Italy (https://www.gisaid.org/) [22], while the C3140T-C295557 variants were highly observed in Portugal by the time of this patient’s infections, especially so around her geographical region [23]. This viral haplotype was also obtained from the RNA extracted from the diagnostic sample of her 41-year-old mother. Transmission is supposed to have occurred from the patient to her mother. Other cohabitants, her 45-year-old father and 8-year-old brother, never presented symptoms, and three and half months later, they had no serological evidence of past SARS-CoV-2 infection. However, her asymptomatic boyfriend showed reactivity to specific anti-SARS-CoV-2 IgG antibodies.

The second serial sample (P1.2), at day nine after diagnosis and coinciding with the first hospital discharge, already revealed a mixed viral profile compatible with a 3% co-infection by a basal 20B affiliated haplotype, which had become highly frequent throughout Europe [24,25]. This 3%-20B haplotype is defined by G28881A-G28882A-G28883C variants and shares C241T-C3037T-C14408T-A23403G variants with the 20A haplotype (hence, these shared variants had 100% frequency in P1.2). Thus, the two infecting haplotypes diverged by six SNPs. As this sample had a low viral load, we repeated the sequencing process twice and confirmed that in both runs amongst different batches of samples, we obtained the same admixed profile of around 3% 20B. We ended up summing the total reads in the reported sequence.

Unfortunately, as April and May matched the peak of the first COVID-19 wave in Portugal, the hospital processed thousands of daily samples, being unable to store them and having to resort to other extraction and RT-PCR kits. So, no samples matching the first period at-home quarantine and second inpatient stay of this patient were stored.

However, we could ascertain that at least 12 days after the second hospital discharge (samples P1.3 to P1.7), the 20B lineage had totally replaced the initially dominant 20A lineage, including a sample with a negative RT-PCR result (P1.5). The samples with lower viral load (P1.2, P1.6, and P1.7) had other variants (Table S4), at variable frequencies, in addition to the ones defining the lineages backbones. The variants that accumulate in the patient samples with lower viral load, or in other words, farther apart from the events that led to hospitalizations, can be due to two factors: (1) a lower resolution of our sequencing; (2) real representatives of the intra-host genomic diversity, and the plasticity of SARS-CoV-2 [26], which is a phenomenon known as quasispecies [27].

For the stored later nasopharyngeal samples, it was impossible to retrieve integral and infectious SARS-CoV-2 viral particles through in vitro culture (Figure S4), despite some of these samples being still positive in the RT-PCR.

### 3.3. Genome-Wide Array Screening and Polygenic Risk Score

**Figure 3:**
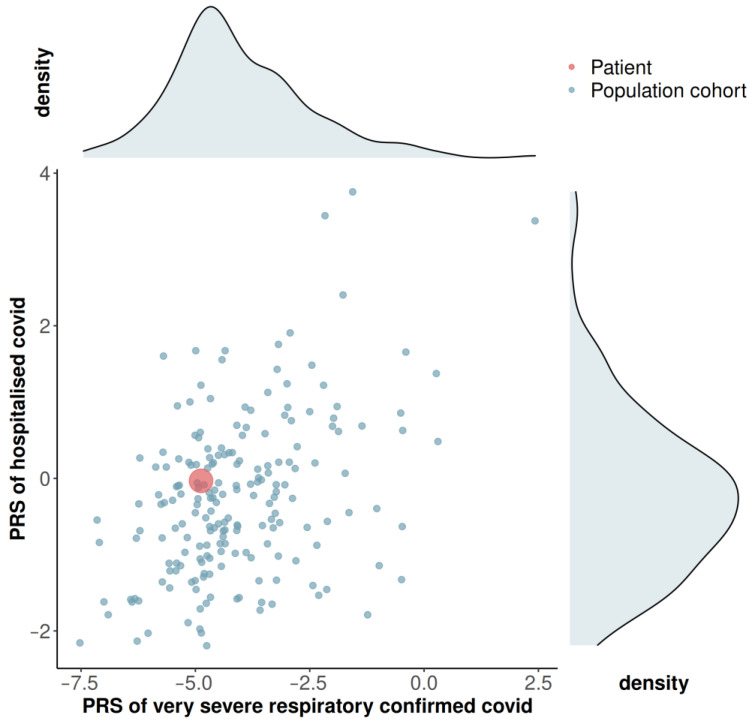
Scatter plot of the polygenic risk score (PRS) values and respective density plots for “very severe respiratory confirmed covid” and “hospitalized covid” phenotypes for the Portuguese population cohort (*n* = 198), and the scatter point value for the patient (in red). The PRS values were calculated based on odd ratios reported online by the COVID-19 host genetics initiative (https://www.covid19hg.org/) [21].

We are certain that these RNA samples belong to the patient, and were not mismatched in the hospital or in the laboratories, through the characterization of an array containing 900 K human variants. The 900 K-profile was shared between the seven samples, and it is unique of the patient, as she has no monozygotic twin. The 900 K genotyping in the patient also allowed estimating the PRS values for “very severe respiratory confirmed covid” and “hospitalized covid”, based on the incipient evidence that is being collected by the COVID-19 host genetics initiative [21]. The patient values were contextualized in a Portuguese population cohort (Figure 3; Tables S5–S7). The PRS values for the patient were within the normal distributions observed in the population cohort, almost matching the mean values.

## 4. Discussions

We illustrate a severe presentation of COVID-19 in a young healthy patient with prolonged viral shedding of SARS-CoV-2. This case was extremely challenging in terms of clinical diagnosis, and only the molecular study allowed us to shed light into its classification as the first proven SARS-CoV-2 co-infection.

The affiliation of the two haplotypes in distinct clades, which emerged at different time points along the pandemic (see https://nextstrain.org/sars-cov-2/) [28], renders it unlikely that they evolved intra-host from one another. The co-infection with a virus belonging to a different clade had to occur between 29th February and 18th March, which is the 19-day period between the beginning of symptoms and detection of the two lineages. However, we hypothesize that co-infection was already present from disease onset, and we simply failed to detect the second lineage in the first diagnostic sample. This time window precedes the production of IgG antibodies by the immune system.

The episode that led to the second hospitalization could be speculated as corresponding to the moment when the 20B lineage became dominant. Unfortunately, we have no samples from this period allowing us to confirm this speculation. It is a fact that the viral load was higher (10–25 times) in the samples after this episode comparing with those from the date of the first discharge. Even so, we found no in vitro evidence that the patient was contagious at this period. These results agree with previous findings [29,30] that a later-on positive RT-PCR does not imply the presence of active viral particles.

All this evidence seems to imply that the early-on co-infection by two SARS-CoV-2 lineages may have contributed to the severe disease displayed by this young and healthy female. Furthermore, the PRS evidence testified that she had no genetic predisposition for hospitalization or severe COVID-19 when comparing with a Portuguese population cohort. The dynamics of exchange between dominant lineages may have contributed to such a prolonged viral shedding case. Most probably, the mother was only infected by one lineage, hence displaying the milder disease, although we could not test it due to the unavailability of samples.

For sure, in the near future, several other cases of co-infection and reinfection will be identified as the pandemic continues. These cases will help clarify if a worse disease course can be caused by the overlapping or sequential infection by different SARS-CoV-2 lineages due to ADE. The authors of the paper reporting the first reinfection in the USA patient [12], who presented with a second infection symptomatically more severe than the first, pointed to the possibility of co-infection instead of reinfection, implying that they simply did not detect overlapping “specimens A and B” in the April 2020 sample. In this case, as the patient was considered recovered after the first hospitalization, he was not isolated, rendering the hypothesis of reinfection more probable. Reinfection would also agree with the more severe second COVID-19 episode presented by this patient. Both this case and the one we present here highlight the importance of performing more molecular studies on virus transmission dynamics. Either way, co-infection or reinfection, taking into account the possibility that the immunity driven by a specific SARS-CoV-2 lineage does not protect against another lineage, but can instead lead to a more severe disease pattern, is of extreme relevance in public health.

## Data Availability

Extensive files listing the variant calls for the viral whole-genome sequences can be downloaded from https://portal.i3s.up.pt/docs/bcavadas/PEDROetal.zip. Given that the genome-wide profiles from the patient samples allow individual identification, researchers can request the data from the corresponding author, after justifying its need for the advancement of research.

## Supplementary Materials

The following are available online at https://www.mdpi.com/2076-2607/9/2/300/s1, Figure S1: X-ray (A) and chest CT-scan (B–G) pictures at the time of COVID-19 diagnosis, Figure S2: Angio-chest CT scan performed after 6 days of inpatient stay, Figure S3: X-ray (A) and chest CT-scan (B–D) pictures from re-admission, Figure S4: Results from the in vitro culture of SARS-CoV-2 from the nasopharyngeal samples, Table S1: Results of the blood tests collected at admission during the 1st inpatient stay (2020-03-10) and at the time of COVID-19 symptom recurrence that led to a 2nd inpatient stay (2020-05-12), Table S2: Results of the microbiologic workup collected at admission during the 1st inpatient stay (2020-03-10) and at the time of COVID-19 symptom recurrence that led to a 2nd inpatient stay (2020-05-12), Table S3: Immunophenotyping of peripheral blood lymphocytes and quantitative immunoglobulins’ test performed during the 2nd inpatient stay, Table S4: List of variants detected in all the analyzed samples with a minimum of 5% frequency in at least one of the samples, Table S5: List of the significantly associated variants used for PRS calculation based on the A2_ALL dataset of “very severe respiratory confirmed covid” (n = 2972) vs. population (n = 284,472), Table S6: List of the significantly associated variants used for PRS calculation based on the B2_ALL dataset of “hospitalized covid” (n = 6492) vs. population (n = 1,012,809), Table S7: PRS score values for the “very severe respiratory confirmed covid” and “hospitalized covid” phenotypes in the patient and individuals from the Portuguese cohort.
