# Supplementary material for: Dynamics of a Dual SARS-CoV-2 Lineage Co-Infection on a Prolonged Viral Shedding COVID-19 Case: Insights into Clinical Severity and Disease Duration
Source: Microorganisms. 2021 Feb 2;9(2):300. doi: 10.3390/microorganisms9020300 (PMC7912897; doi:10.3390/microorganisms9020300)
Supplement: Supplementary file 1 [file microorganisms-09-00300-s001.pdf]

Dynamics of a dual SARS-CoV-2 strain co-infection on a prolonged viral shedding COVID-19 case: insights into clinical severity and disease duration

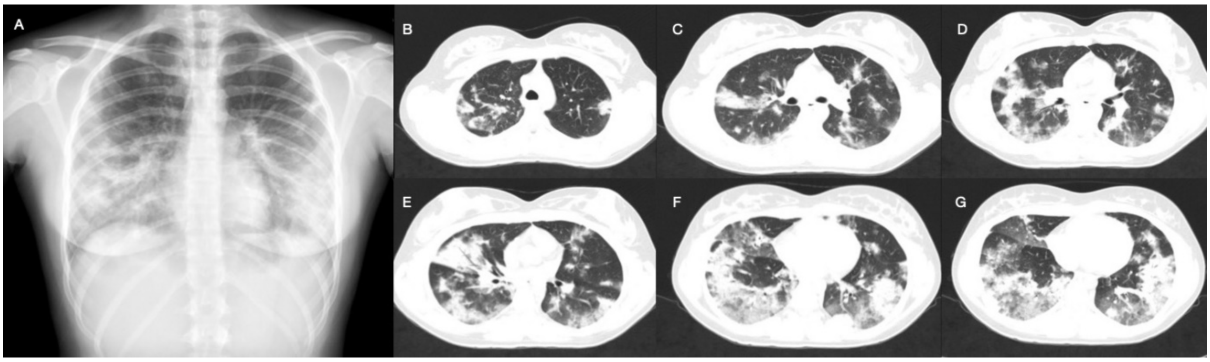

Supplementary Figure S1. X-ray **(A)** and chest CT-scan **(B-G)** pictures at the time of COVID-19 diagnosis (2020-03-10) revealing extensive bilateral subpleural ground-glass opacities (GGO) with areas of air-space consolidation concerning for COVID-19.

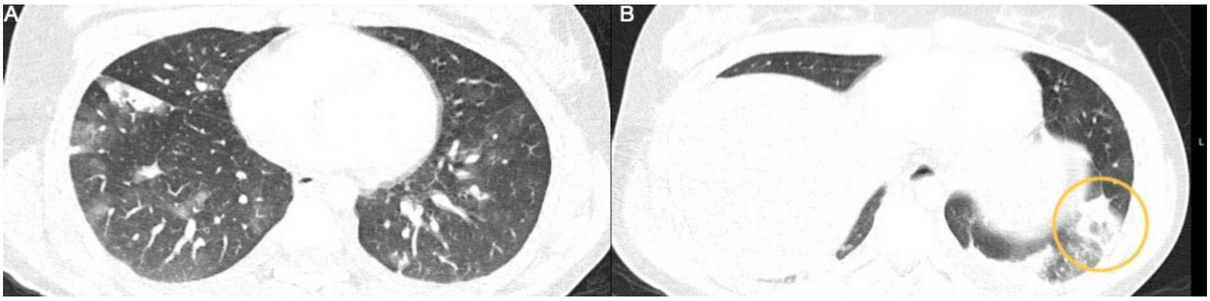

Supplementary Figure S2. Angio-chest CT scan performed after 6 days of inpatient stay (2020-03-16). Multiple foci of peripheral GGO and alveolar consolidation **(A)**. Heterogeneous ground-glass opacification with a peripheral halo of consolidation suggestive of pulmonary infarction of the left lung lobe (encircled in picture **B**).

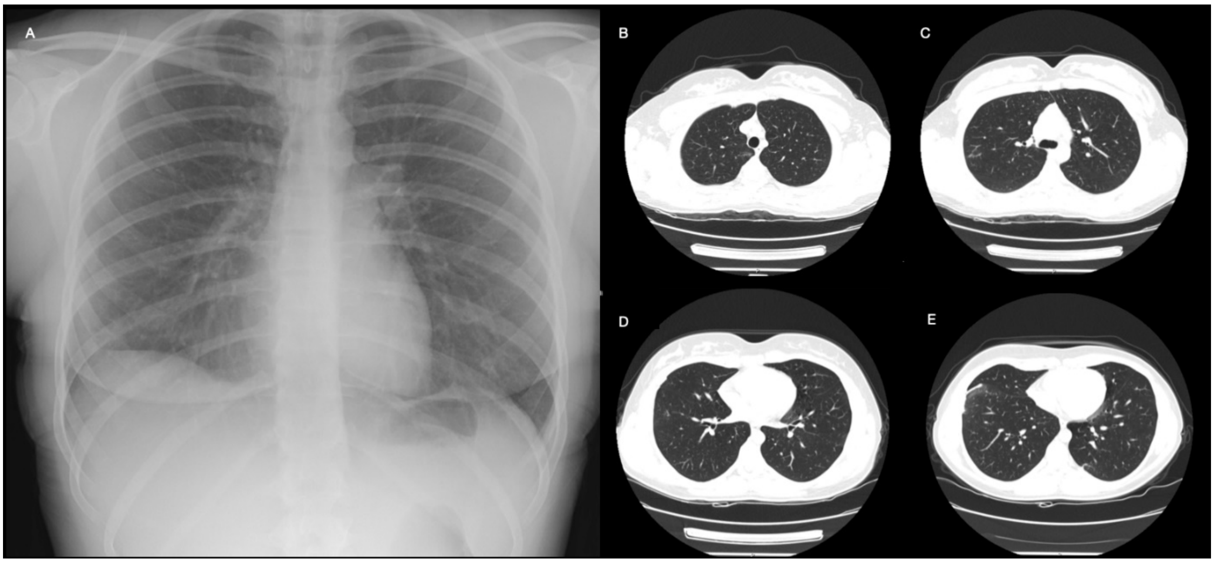

Supplementary Figure S3. X-ray **(A)** and chest CT-scan **(B-D)** pictures from re-admission (2020-05-12), nearly 2 months after COVID-19 diagnosis, revealing improved aeration of the lung fields and resolving features of ground-glass-opacities.

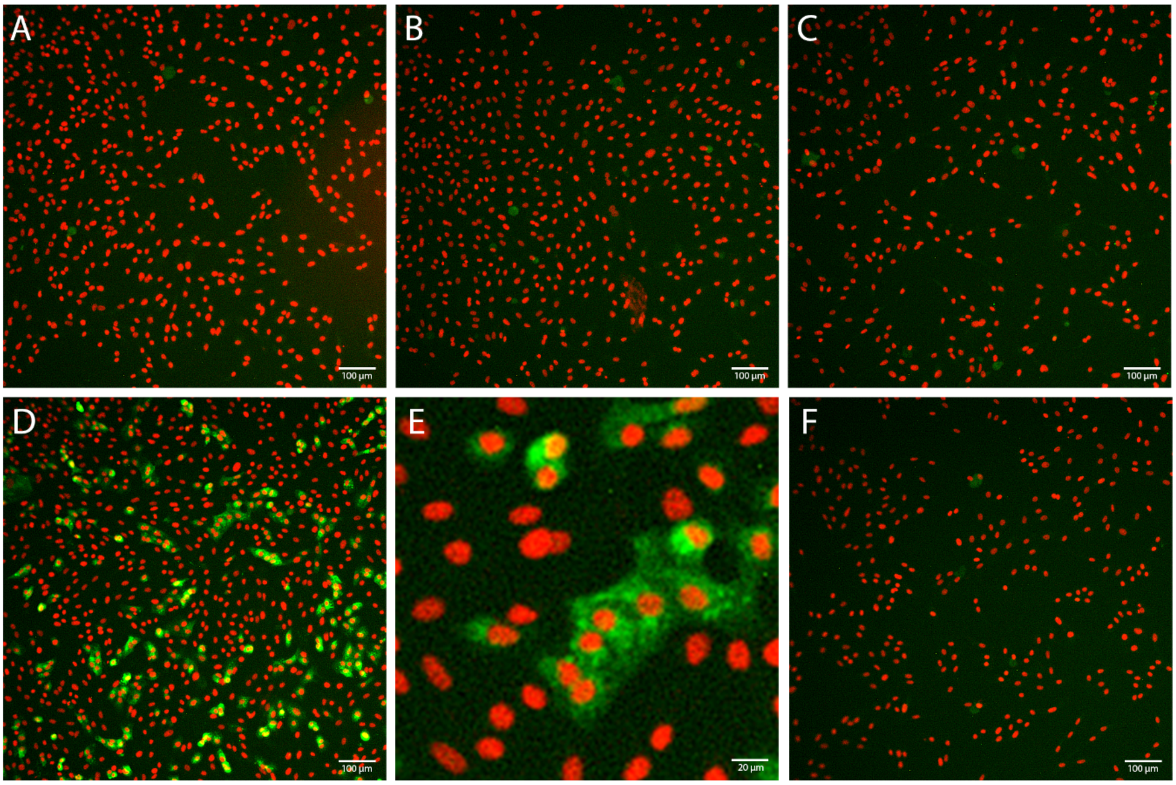

Supplementary Figure S4. Results from the in vitro culture of SARS-CoV-2 from the nasopharyngeal samples. Images from immunofluorescence staining with the antibody against SARS-CoV-2 spike protein (green) and the marker for the nucleus (red), at 48h after the second inoculation in Vero cells. Images were acquired in an IN Cell Analyzer 2000, with 10x objective. Negative results in samples from the patient at May 26th (A), June 9th (B) and June 12th (C). Positive result (D) and higher detail (E) for a recently-diagnosed sample from another patient. Negative result (F) for control of non-infected cells. Bars represent 100µm or 20µm.

Supplementary Table S1. Results of the blood tests collected at admission during the 1st inpatient stay (2020-03-10) and at the time of COVID-19 symptom recurrence that led to a 2nd inpatient stay (2020-05-12). AST – Aspartate aminotransferase; ALT- Alanine Aminotransferase; GGT- Gamma-glutamyl-transferase; ALP– Alkaline Phosphatase; LDH – Lactate Dehydrogenase; CK – Creatinine Kinase; hs-cTNI – High-sensitivity Troponin I; CK-MB- Creatine Kinase MB; aPTT- Activated Partial Thromboplastin Time; PT- Prothrombin Time; CRP- C-reative-protein; NP- not performed.

Blood tests	1st inpatient stay results at admission (2020-03-10)	2nd inpatient stay results at admission (2020-05-12)	Normal range values
**Haemoglobin (g/dL)**	14.2	14.7	12.0-16.0
**Leukocytes (x 10^9/L)**	5.7	6.7	4.0-11.0
**Neutrophils (%)**	82.6	58.2	53.8-69.8
**Lymphocytes (%)**	12.9	34.7	22.6-36.6
**Platelets (x 10^9/L)**	172	290	150-400
**AST (U/L)**	34	19	10-31
**ALT (U/L)**	24	24	10-31
**GGT (U/L)**	41	NP	7-32
**ALP (U/L)**	50	NP	47-119
**Total bilirubin (mg/dL)**	0.60	NP	< 1.20
**Direct bilirubin (mg/dL)**	0.16	NP	< 0.40
**LDH (U/L)**	325	NP	135-225
**CK (U/L)**	74	NP	10-149
**hs-cTnI (ng/L)**	< 1.9	< 1.9	< 16.0
**CK-MB (ng/mL)**	0.30	0.30	0.00-6.40
**Mioglobin (ng/mL)**	21.5	20.2	< 146.9
**Urea (mg/dL)**	21	20	10-50
**Plasma creatinine (mg/dL)**	0.57	0.80	0.51-0.95
**aPTT (sec)**	27.2	30.7	24.2-36.4
**PT (sec)**	12.7	11.9	9.9-13.6
**Fibrinogen (mg/dL)**	382	248	200-400
**CRP (mg/L)**	37.6	0.40	< 3.0
**Ferritin (ng/mL)**	NP	76.28	4.63-204

Supplementary Table S2. Results of the microbiologic workup collected at admission during the 1st inpatient stay (2020-03-10) and at the time of COVID-19 symptom recurrence that led to a 2nd inpatient stay (2020-05-12). Ag – Antigen; NAAT – Nucleic Acid Amplification Test; NP- not performed.

	Product	1st inpatient stay results (2020-03-10)	2nd inpatient stay results (2020-05-12)
Blood cultures (each pair)	Blood	negative	negative
Bacterial cultures	Sputum	negative	negative
Pneumococcal Ag	Urine	negative	negative
Legionella Ag	Urine	negative	negative
Bordetella pertussis NAAT	Naso/oropharyngeal swab	negative	negative
Chlamydia pneumoniae NAAT	Naso/oropharyngeal swab	negative	negative
Mycoplasma pneumoniae NAAT	Naso/oropharyngeal swab	negative	negative
Adenovirus NAAT	Naso/oropharyngeal swab	negative	negative
Coronavirus 229E, HKU1, NL63, OC43 NAAT	Naso/oropharyngeal swab	negative	negative
Metapneumovirus NAAT	Naso/oropharyngeal swab	negative	negative
Rhinovirus/Enterovirus NAAT	Naso/oropharyngeal swab	negative	negative
Influenza A & B NAAT	Naso/oropharyngeal swab	negative	NP
Parainfluenza 1,2,3 & 4 NAAT	Naso/oropharyngeal swab	negative	negative
Bocavirus NAAT	Naso/oropharyngeal swab	NP	negative
**SARS-CoV-2 NAAT**	**Naso/oropharyngeal swab**	**positive**	**positive**

Supplementary Table S3. Immunophenotyping of peripheral blood lymphocytes and quantitative immunoglobulins' test performed during the 2nd inpatient stay (2020-05-12).

**Immunophenotyping of peripheral blood lymphocytes**	**Results**
**Lymphocytes (flow cytometry)**	3059/mm^3
**T populations:**	
CD3+	78.93%
CD3+CD4+	48.83%
(absolute value)	1494/mm^3
CD3+CD8+	27.19%
CD3+CD4+CD8+	0.33%
Ratio CD4+/CD8+	1.80
**B populations:**	
CD19+	9.02%
**NK cell populations:**	
CD16&56+	11.62%
**Immunoglobulins** (normal range values)	**Results**
**Immunoglobulin G** (650-1500 mg/dL)	955
**IgG subclasses:**	
IgG1 (370.0-1280.0 mg/dL)	705.0
IgG2 (106.0-610.0 mg/dL)	284.0
IgG3 (18.0-163.0 mg/dL)	42.6
IgG4 (4.0-230.0 mg/dL)	17.0
**Immunoglobulin A** (78-312 mg/dL)	137
**Immunoglobulin M** (55-300 mg/dL)	127

Supplementary Table S4. List of variants detected in all the analysed samples with a minimum of 5% frequency in at least one of the samples. The positions painted in blue are represented in Figure 3 and the grey coloured cells are the positions with variation.

POS	REF	ALT	11/03/2020 Mother of P1		10/03/2020 P1.1		18/03/2020 P1.2		26/05/2020 P1.3		06/06/2020 P1.4		09/06/2020 P1.5		11/06/2020 P1.6		12/06/2020 P1.7	
			N reads	Avg het	N reads	Avg het	N reads	Avg het	N reads	Avg het	N reads	Avg het	N reads	Avg het	N reads	Avg het	N reads	Avg het
**72**	G	A	17979	0.0	33542	0.0	182	0.0	723	0.0	14180	0.0	74	0.0	942	54.6	20	0.0
**128**	T	C	13357	0.0	21817	0.0	7	0.0	20	0.0	2306	10.8	31	0.0	0	0.0	7	0.0
**144**	T	C	10366	0.0	20117	0.0	6	0.0	18	0.0	1999	11.8	24	0.0	0	0.0	6	0.0
**186**	C	T	11906	0.0	21162	0.0	8	0.0	18	0.0	2204	10.7	22	0.0	0	0.0	5	0.0
**241**	C	T	7627	100.0	16314	100.0	4	100.0	15	100.0	1422	100.0	4	100.0	0	0.0	0	0.0
**262**	T	C	11543	0.0	28796	0.0	13	0.0	29	0.0	3964	5.4	273	0.0	0	0.0	7	0.0
**338**	G	A	23813	0.0	43641	0.0	16	0.0	57	0.0	4718	6.0	13	0.0	1139	0.0	7	0.0
**592**	T	C	12027	0.0	38376	0.0	673	0.0	415	0.0	2560	9.0	408	0.0	0	0.0	22	0.0
**621**	G	A	12233	0.0	38804	0.0	679	0.0	414	0.0	2568	13.1	128	0.0	0	0.0	21	0.0
**1204**	C	T	8429	0.0	16516	0.0	6	0.0	43	0.0	3652	5.8	3	0.0	109	0.0	3	0.0
**1367**	A	G	5997	5.8	21891	0.0	9	0.0	39	0.0	4513	0.0	7	0.0	477	0.0	3	0.0
**1459**	T	C	10715	0.0	17352	0.0	7	42.9	32	0.0	3855	8.3	5	0.0	23	0.0	4	0.0
**1745**	T	C	5655	0.0	21029	0.0	4	0.0	18	0.0	563	0.0	1	0.0	2	0.0	2	100.0
**1895**	G	A	7627	0.0	26589	0.0	19	0.0	46	0.0	6302	0.0	6	0.0	0	0.0	9	33.3
**2013**	C	T	6990	0.0	30450	0.0	7	0.0	66	0.0	1349	11.8	11	0.0	295	0.0	66	0.0
**2217**	T	C	7873	0.0	29341	0.0	8	0.0	14	0.0	2895	6.4	15	0.0	18	0.0	11	0.0
**2240**	A	G	5786	0.0	27623	0.0	7	0.0	15	0.0	2489	7.3	13	0.0	13	0.0	10	0.0
**2419**	A	G	3638	0.0	15566	0.0	199	0.0	9	0.0	1410	8.5	10	0.0	12	0.0	6	0.0
**2501**	A	G	2556	0.0	13809	0.0	308	40.3	2	0.0	1413	0.0	4	0.0	75	0.0	9	0.0
**2818**	A	G	10039	0.0	36617	0.2	250	0.0	246	0.0	9009	0.0	9	0.0	1503	33.5	8	0.0
**2901**	T	C	16112	0.0	48138	0.3	134	5.2	1583	0.0	52328	2.0	26	0.0	65	0.0	25	0.0
**2903**	A	G	16016	0.0	47839	0.0	130	5.4	1595	0.0	52781	0.0	27	0.0	66	0.0	25	0.0
**3037**	C	T	10924	100.0	32832	100.0	201	100.0	1263	100.0	44978	100.0	5	100.0	3	100.0	18	100.0
**3140**	C	T	5925	99.9	19494	100.0	167	88.0	43	0.0	5805	0.0	2	0.0	1	0.0	26	0.0
**3477**	T	C	3917	0.0	8584	0.0	0	0.0	19	0.0	1365	5.4	1	0.0	1	0.0	0	0.0
**3512**	A	G	6594	0.0	17428	0.0	637	41.3	68	0.0	5492	0.0	6	0.0	1	0.0	8	0.0
**3514**	G	A	6561	0.0	17458	0.0	637	0.0	66	0.0	5525	7.3	5	0.0	1	0.0	8	0.0
**3667**	T	C	6776	0.0	20763	0.0	328	96.3	36	0.0	3371	0.0	5	0.0	83	0.0	6	0.0
**4411**	A	G	3685	0.0	18706	0.0	703	45.1	24	0.0	11	0.0	5	0.0	2	0.0	8	0.0
**4535**	T	C	7106	0.0	27151	0.3	181	0.0	34	0.0	6839	6.2	7	0.0	440	0.0	9	0.0
**4543**	C	T	9963	0.0	34131	0.0	230	54.8	54	0.0	8636	0.0	5	0.0	578	0.0	12	0.0
**4672**	C	T	1804	8.2	16600	0.0	5	0.0	12	0.0	7	0.0	3	0.0	1	0.0	5	0.0
**4673**	A	G	1320	6.1	15444	0.0	3	0.0	7	0.0	7	0.0	4	0.0	0	0.0	3	0.0
**4759**	A	G	6147	0.0	17337	0.0	70	0.0	148	0.0	7806	6.9	6	0.0	0	0.0	121	0.0
**4956**	A	G	6053	0.0	25312	0.0	307	0.0	66	0.0	2935	8.0	17	0.0	195	0.0	54	0.0
**5030**	A	G	5493	0.0	14940	0.0	341	0.0	37	0.0	3150	0.0	14	0.0	213	7.0	10	0.0
**5055**	C	T	5586	0.0	14608	0.0	339	0.0	35	0.0	2471	17.6	2	0.0	221	0.0	5	0.0

POS	REF	ALT	11/03/2020 Mother of P1		10/03/2020 P1.1		18/03/2020 P1.2		26/05/2020 P1.3		06/06/2020 P1.4		09/06/2020 P1.5		11/06/2020 P1.6		12/06/2020 P1.7	
			N reads	Avg het	N reads	Avg het	N reads	Avg het	N reads	Avg het	N reads	Avg het	N reads	Avg het	N reads	Avg het	N reads	Avg het
**5199**	G	A	3703	0.0	12476	0.0	325	0.0	21	0.0	1895	11.4	1	0.0	2	0.0	2	0.0
**5325**	A	G	3861	0.0	9117	0.0	319	0.0	16	0.0	2044	7.5	0	0.0	25	0.0	1	0.0
**5896**	A	G	4374	0.0	16370	0.0	6	0.0	12	0.0	3013	0.0	2	0.0	29	0.0	3	66.7
**5921**	G	A	6482	0.0	18894	0.0	10	0.0	28	0.0	3745	0.0	0	0.0	35	28.6	4	0.0
**5984**	T	A	421	6.4	8061	0.0	0	0.0	4	0.0	233	0.0	1	0.0	0	0.0	4	0.0
**6170**	G	A	8993	0.0	30990	0.0	54	0.0	27	0.0	1652	11.1	6	0.0	258	0.0	10	0.0
**6198**	C	T	9860	0.0	32941	0.0	56	0.0	27	0.0	1821	0.0	6	0.0	262	5.7	11	0.0
**6200**	T	C	10871	0.0	34092	0.0	59	0.0	29	0.0	1950	9.4	7	0.0	273	0.0	11	0.0
**6238**	T	C	11250	0.0	34647	0.0	56	85.7	31	0.0	2016	0.0	9	0.0	284	0.0	12	0.0
**6254**	G	A	773	5.4	13265	0.0	55	0.0	12	0.0	164	0.0	6	0.0	292	0.0	8	0.0
**6475**	A	G	8374	0.0	23484	0.0	381	0.0	46	0.0	1536	12.4	17	0.0	0	0.0	9	0.0
**6675**	C	T	6242	0.0	31757	0.0	414	0.0	472	0.0	14436	12.4	12	0.0	1156	0.0	304	0.0
**6701**	C	T	6032	0.0	31314	0.0	407	0.0	463	0.0	14034	2.6	12	0.0	1113	11.8	313	0.0
**6755**	G	A	2079	0.0	18680	11.5	273	0.0	92	0.0	1742	0.0	7	0.0	566	0.0	279	0.0
**6882**	T	C	3205	0.0	8204	0.0	91	0.0	3	0.0	385	5.5	0	0.0	0	0.0	0	0.0
**6920**	A	G	3079	0.0	14260	0.0	166	0.0	10	0.0	370	9.5	4	0.0	0	0.0	3	0.0
**6958**	T	C	2343	0.0	14369	0.0	159	0.0	10	0.0	337	11.9	5	0.0	0	0.0	5	0.0
**6982**	C	T	2374	0.0	14384	0.3	153	0.0	8	0.0	327	8.0	5	0.0	0	0.0	5	0.0
**6991**	T	C	1657	0.0	12657	0.0	122	0.0	8	0.0	265	6.4	5	0.0	0	0.0	3	0.0
**7225**	T	C	1586	10.6	10328	0.6	21	0.0	4	0.0	737	0.0	1	0.0	0	0.0	1	0.0
**7303**	C	T	311	0.0	7085	12.1	43	0.0	1	0.0	0	0.0	4	0.0	0	0.0	4	0.0
**7359**	T	C	4327	0.0	17263	0.0	50	0.0	15	0.0	2459	0.0	12	33.3	0	0.0	26	19.2
**7513**	T	C	4487	0.0	19847	0.0	12	0.0	26	0.0	3676	5.1	5	0.0	1	0.0	18	0.0
**7537**	A	G	3878	0.0	19631	0.0	11	0.0	20	0.0	3474	5.0	3	0.0	1	0.0	13	0.0
**8193**	A	G	3485	7.3	15758	0.0	8	0.0	14	0.0	3050	0.0	5	0.0	246	0.0	9	0.0
**8388**	A	G	4947	0.0	18886	0.0	525	41.7	522	0.0	24309	0.0	9	0.0	1050	0.0	9	0.0
**8389**	C	T	4877	5.7	18899	0.0	511	0.0	529	0.0	23956	0.0	8	0.0	1008	0.0	9	0.0
**8750**	A	G	4464	3.9	19109	0.0	371	0.0	92	0.0	9222	0.0	8	0.0	388	5.7	6	0.0
**8928**	T	G	2626	4.7	12005	0.0	153	5.9	20	0.0	3965	2.3	3	0.0	259	0.0	3	0.0
**9071**	A	G	8749	0.0	25287	0.0	37	0.0	897	0.0	21944	0.4	5	0.0	831	34.1	21	0.0
**9481**	T	C	2743	0.0	13519	0.0	3	66.7	28	0.0	191	0.0	11	0.0	718	0.0	12	0.0
**9704**	T	C	2870	0.0	14830	0.0	2	0.0	8	0.0	473	5.9	5	0.0	0	0.0	2	0.0
**10024**	A	G	9789	0.0	27393	0.0	215	0.0	88	0.0	8892	0.0	2	0.0	1377	24.6	24	0.0
**10201**	G	A	7377	0.0	30149	0.0	169	0.0	78	0.0	7613	5.2	16	0.0	2	0.0	8	0.0
**10389**	T	C	4577	0.0	26143	0.0	616	0.0	84	0.0	11059	0.0	11	0.0	462	47.8	32	0.0
**10409**	A	G	4214	0.0	27736	0.0	589	0.0	70	0.0	10855	11.2	7	0.0	426	0.0	32	0.0
**10411**	T	C	5048	0.0	28279	0.0	625	0.0	88	0.0	11313	16.0	10	0.0	487	0.0	32	0.0
**10549**	G	A	4954	0.0	24894	0.0	284	0.0	29	0.0	3900	6.7	3	0.0	637	0.0	5	0.0
**10622**	A	C	1942	0.0	9178	0.0	81	9.9	20	0.0	50	0.0	2	0.0	277	0.0	7	0.0
**10729**	A	G	3480	0.0	25543	0.0	156	0.0	54	0.0	3701	6.3	6	0.0	1	0.0	13	0.0

POS	REF	ALT	11/03/2020 Mother of P1		10/03/2020 P1.1		18/03/2020 P1.2		26/05/2020 P1.3		06/06/2020 P1.4		09/06/2020 P1.5		11/06/2020 P1.6		12/06/2020 P1.7	
			N reads	Avg het	N reads	Avg het	N reads	Avg het	N reads	Avg het	N reads	Avg het	N reads	Avg het	N reads	Avg het	N reads	Avg het
**10763**	T	C	4324	0.0	25689	0.0	168	0.0	81	0.0	4000	8.4	8	0.0	1	0.0	19	0.0
**10973**	A	G	3434	0.0	11312	0.0	270	9.6	15	0.0	5189	0.0	1	0.0	1	0.0	2	0.0
**11076**	T	C	4112	6.1	17403	0.0	273	0.0	15	0.0	4121	0.0	1	0.0	1	0.0	3	0.0
**11083**	G	T	4935	2.6	24865	0.9	119	89.9	18	0.0	5368	0.0	1	0.0	2	0.0	3	0.0
**11180**	T	C	3694	0.0	14495	0.0	143	0.0	28	0.0	1430	15.1	1	0.0	0	0.0	2	0.0
**11185**	G	A	6954	0.0	26870	0.0	259	0.0	45	0.0	2553	0.0	5	0.0	3	100.0	6	0.0
**11243**	G	A	4602	0.0	12203	0.0	196	0.0	29	0.0	2019	12.5	0	0.0	0	0.0	3	0.0
**11454**	C	T	5221	0.0	20942	0.0	48	0.0	36	0.0	3112	0.0	9	0.0	712	15.3	9	0.0
**11638**	T	C	2138	0.0	15689	0.0	164	0.0	22	0.0	316	100.0	4	0.0	0	0.0	16	0.0
**11827**	A	G	13786	0.0	56716	0.0	20	15.0	144	0.0	10927	5.7	8	0.0	2799	0.0	14	0.0
**11851**	G	A	17115	0.0	58806	0.0	26	19.2	182	0.0	12998	19.1	6	0.0	3057	0.0	17	0.0
**11968**	T	C	10865	0.0	42559	0.0	17	0.0	109	0.0	5742	0.0	8	0.0	2463	15.7	18	0.0
**12519**	A	G	7656	0.0	27051	0.0	371	0.0	100	0.0	3789	0.0	12	33.3	0	0.0	8	0.0
**12613**	G	A	9133	0.0	41789	0.0	86	0.0	142	0.0	3083	6.5	21	0.0	1	0.0	23	0.0
**12652**	A	G	9938	0.0	42570	0.0	90	0.0	158	0.0	3157	5.9	25	0.0	0	0.0	24	0.0
**12670**	T	C	10092	0.0	42207	0.0	92	0.0	155	0.0	3168	0.0	23	17.4	1	0.0	23	0.0
**13401**	A	G	9064	0.0	20811	0.0	416	0.0	102	0.0	4535	5.1	46	0.0	283	0.0	278	0.0
**13415**	G	A	9137	0.0	21132	0.0	413	0.0	105	0.0	4597	0.0	49	0.0	291	0.0	282	9.9
**13427**	G	A	9811	0.0	21147	0.0	420	0.0	105	0.0	4712	0.0	48	0.0	284	6.7	280	0.0
**13470**	G	A	8206	0.0	17507	0.0	309	0.0	11	0.0	3583	6.3	5	0.0	0	0.0	0	0.0
**13616**	A	G	3452	0.0	12825	0.0	0	0.0	7	0.0	779	12.7	0	0.0	0	0.0	2	0.0
**13875**	A	G	8103	0.0	24955	0.0	10	0.0	27	0.0	1192	16.4	5	0.0	228	0.0	3	0.0
**14006**	C	A	8841	0.0	33679	0.0	12	0.0	77	0.0	3682	5.0	11	0.0	703	0.0	7	0.0
**14188**	G	A	6957	6.2	27983	0.0	6	0.0	45	0.0	3369	0.0	12	0.0	254	0.0	13	0.0
**14408**	C	T	445	100.0	9840	100.0	0	0.0	3	66.7	86	0.0	187	100.0	921	100.0	3	100.0
**14424**	A	G	984	0.0	10483	0.0	26	0.0	186	0.0	10411	7.3	191	0.0	942	0.0	4	0.0
**14530**	T	C	2463	5.3	14456	0.0	45	0.0	259	0.0	17223	0.0	5	0.0	1033	0.0	20	0.0
**14586**	T	C	2009	0.0	14203	0.0	1	0.0	41	0.0	1	0.0	6	0.0	746	0.0	20	40.0
**14846**	T	C	5465	0.0	18377	0.0	135	14.1	18	0.0	2702	0.0	5	0.0	0	0.0	8	0.0
**14857**	G	A	4915	0.0	18510	0.0	136	5.2	15	0.0	2587	0.0	6	0.0	0	0.0	8	0.0
**14899**	T	C	1033	0.0	7699	0.0	140	0.0	14	0.0	43	39.5	2	0.0	0	0.0	4	0.0
**14913**	C	T	994	0.0	7670	0.0	134	0.0	24	33.3	336	0.0	1	0.0	0	0.0	3	0.0
**15189**	A	G	13945	0.0	36581	0.2	299	0.0	63	0.0	5393	0.0	19	0.0	312	0.0	15	40.0
**15201**	A	G	14150	0.0	36618	0.0	300	0.0	61	0.0	5381	0.0	17	0.0	322	0.0	15	33.3
**15444**	G	A	24934	0.0	32766	0.0	486	11.1	390	0.0	15905	0.0	16	0.0	0	0.0	10	0.0
**15570**	T	C	30872	0.0	62167	0.0	484	10.3	694	0.0	16112	0.0	237	0.0	1389	0.0	24	0.0
**15725**	C	T	18173	0.0	40821	0.0	10	0.0	364	0.0	3109	0.0	264	0.0	1482	0.0	22	13.6
**15758**	A	G	9238	0.0	10576	0.0	8	0.0	9	0.0	1871	7.1	12	0.0	0	0.0	2	0.0
**15804**	T	C	11603	0.0	17539	0.0	177	0.0	26	0.0	4053	0.0	11	54.6	0	0.0	5	0.0
**15851**	A	C	1267	1.7	7582	0.8	47	14.9	7	0.0	1076	0.0	3	0.0	1	0.0	1	0.0

POS	REF	ALT	11/03/2020 Mother of P1		10/03/2020 P1.1		18/03/2020 P1.2		26/05/2020 P1.3		06/06/2020 P1.4		09/06/2020 P1.5		11/06/2020 P1.6		12/06/2020 P1.7	
			N reads	Avg het	N reads	Avg het	N reads	Avg het	N reads	Avg het	N reads	Avg het	N reads	Avg het	N reads	Avg het	N reads	Avg het
**15854**	T	A	1080	7.0	7369	4.2	48	14.6	6	0.0	1001	0.0	3	0.0	1	0.0	0	0.0
**15856**	A	G	1300	5.5	7489	3.5	46	15.2	8	37.5	1074	0.0	4	0.0	1	0.0	1	0.0
**15982**	G	A	14104	1.2	20818	1.4	67	1.5	372	0.0	12298	0.0	9	0.0	4	100.0	14	0.0
**15988**	A	G	5195	0.0	8171	0.0	16	0.0	180	0.0	5409	6.2	3	0.0	2	0.0	3	0.0
**16006**	A	G	15372	0.0	14953	0.3	29	13.8	484	0.0	13590	5.6	3	0.0	3	0.0	12	0.0
**16385**	G	A	18490	0.0	49697	0.0	444	0.0	839	0.0	5551	5.3	15	0.0	4796	24.9	24	0.0
**16424**	T	C	15054	0.6	46381	0.4	432	0.0	757	0.0	4983	0.0	14	0.0	4425	21.0	23	0.0
**16548**	T	C	10605	0.0	30434	0.0	238	0.0	49	0.0	5393	0.0	8	0.0	305	70.2	6	0.0
**16801**	A	T	6649	0.0	20543	0.0	70	0.0	21	0.0	7907	13.6	6	0.0	255	0.0	4	0.0
**17121**	A	G	1798	0.0	14184	0.0	9	0.0	34	0.0	1384	0.0	6	0.0	320	100.0	64	0.0
**17164**	T	C	24748	0.0	47086	0.2	681	0.0	967	0.0	35927	0.0	9	0.0	357	0.0	63	52.4
**17335**	A	G	4089	0.0	19583	0.0	12	0.0	112	0.0	2428	0.0	34	11.8	1	0.0	6	0.0
**17483**	C	T	5757	0.0	10435	0.0	277	0.0	51	0.0	2759	6.3	5	0.0	144	0.0	5	0.0
**17485**	C	T	5685	0.0	10459	0.0	269	0.0	53	0.0	2731	6.1	5	0.0	143	0.0	4	0.0
**17927**	C	T	7383	0.0	23691	0.0	115	0.0	23	0.0	5157	6.1	7	0.0	0	0.0	6	0.0
**18342**	T	C	8198	0.0	28712	0.0	373	0.0	31	71.0	5169	0.0	6	0.0	214	0.0	2	0.0
**18688**	T	C	523	0.0	7635	0.7	210	6.7	5	0.0	3	0.0	1	0.0	1	0.0	1	0.0
**18747**	C	T	3583	0.0	16366	0.0	383	0.0	464	0.0	4919	26.2	3	0.0	66	0.0	68	0.0
**19087**	T	C	6952	0.0	23103	0.0	24	0.0	21	0.0	4127	0.0	4	0.0	1257	0.0	41	34.2
**19287**	T	C	8400	0.0	13344	0.0	170	0.0	13	0.0	2963	7.3	2	0.0	131	0.0	6	0.0
**19521**	T	C	7676	0.0	20076	0.0	130	0.0	37	0.0	1737	42.0	6	0.0	1	0.0	9	0.0
**19819**	C	T	4254	0.0	13493	0.0	209	6.2	21	0.0	2727	0.0	6	0.0	199	0.0	3	0.0
**20262**	A	G	334	6.6	8613	0.0	52	0.0	3	0.0	3	0.0	8	0.0	0	0.0	3	0.0
**20988**	T	C	3484	0.0	17926	0.4	539	5.0	78	0.0	1878	0.0	69	0.0	0	0.0	23	0.0
**21171**	A	G	7670	0.0	21630	0.0	250	9.2	145	0.0	11495	0.0	3	0.0	461	0.0	4	0.0
**21232**	G	A	4397	0.0	22092	0.0	13	0.0	497	0.0	1860	21.6	277	0.0	696	0.0	562	0.0
**21429**	T	A	1078	0.0	13512	0.0	6	0.0	9	0.0	6	66.7	2	0.0	0	0.0	3	0.0
**21765**	T	C	5410	0.0	24104	0.0	23	0.0	24	0.0	13373	0.0	7	0.0	39	10.3	15	0.0
**21788**	A	G	5632	0.0	24110	0.0	23	26.1	26	0.0	13483	23.0	7	0.0	39	0.0	14	0.0
**21908**	T	C	6908	0.0	29762	0.0	24	0.0	62	0.0	2568	5.8	10	0.0	604	0.0	18	0.0
**22025**	A	G	1533	0.0	16354	0.2	5	0.0	30	0.0	198	0.0	5	0.0	539	43.6	7	0.0
**22396**	A	G	4378	0.0	13868	0.0	195	0.0	34	0.0	1502	15.5	1	0.0	180	0.0	24	0.0
**23086**	C	T	5537	5.1	11337	0.0	9	0.0	31	0.0	3556	0.0	4	0.0	146	0.0	8	0.0
**23163**	T	C	5578	0.0	19864	0.2	191	0.0	26	0.0	2817	0.0	8	0.0	451	0.0	15	46.7
**23403**	A	G	6427	100.0	36321	100.0	26	100.0	359	100.0	12792	100.0	6	100.0	475	100.0	17	100.0
**23693**	T	C	7840	0.0	38343	0.2	27	0.0	419	0.0	6470	0.4	109	0.0	5122	0.0	27	14.8
**23698**	T	C	6269	0.0	36625	0.0	24	0.0	316	0.0	6112	0.0	84	0.0	4220	22.9	22	0.0
**23704**	A	G	7788	0.0	37152	0.0	28	0.0	390	0.0	6443	0.0	97	0.0	4742	17.0	27	0.0
**23884**	A	G	2928	0.0	18161	0.0	6	0.0	18	0.0	1071	7.8	5	0.0	0	0.0	6	0.0
**24077**	G	T	5387	100.0	25846	100.0	36	16.7	159	0.0	15747	7.3	7	0.0	694	0.0	17	0.0

POS	REF	ALT	11/03/2020 Mother of P1		10/03/2020 P1.1		18/03/2020 P1.2		26/05/2020 P1.3		06/06/2020 P1.4		09/06/2020 P1.5		11/06/2020 P1.6		12/06/2020 P1.7	
			N reads	Avg het	N reads	Avg het	N reads	Avg het	N reads	Avg het	N reads	Avg het	N reads	Avg het	N reads	Avg het	N reads	Avg het
**24104**	G	A	5552	0.0	26157	0.0	34	0.0	162	0.0	15723	0.0	7	0.0	698	0.0	18	22.2
**24161**	G	A	6292	0.0	26151	0.0	25	0.0	75	0.0	7779	0.4	3	0.0	392	37.2	19	0.0
**24447**	T	C	7455	0.0	22767	0.0	30	0.0	620	0.0	14751	0.0	9	0.0	933	54.5	10	0.0
**24858**	G	A	4626	0.0	25986	0.0	320	0.0	35	0.0	3529	6.9	15	0.0	428	0.0	1	0.0
**24873**	T	C	5507	0.0	25568	0.0	352	0.0	42	0.0	3816	6.7	15	0.0	474	0.0	1	0.0
**24993**	A	G	5618	0.0	27328	0.0	340	0.0	44	0.0	4369	0.0	12	0.0	466	33.7	2	0.0
**25046**	C	T	6197	0.0	28442	0.0	341	0.0	46	0.0	4588	18.2	11	0.0	477	0.0	4	0.0
**25105**	A	G	1001	0.0	6695	0.0	2	0.0	9	0.0	575	7.1	0	0.0	0	0.0	0	0.0
**25440**	G	A	10726	0.0	36120	0.0	17	0.0	260	0.0	7624	2.7	12	0.0	355	80.9	16	0.0
**25556**	T	C	10491	0.0	35154	0.3	447	0.0	1068	0.0	6698	7.7	25	0.0	3137	0.0	1087	12.5
**25608**	A	G	12114	0.0	37929	0.2	894	49.2	2183	0.0	3470	0.0	35	0.0	5303	0.0	1878	0.0
**25657**	A	G	11923	0.0	23571	0.0	52	0.0	77	0.0	13607	8.5	4	0.0	3	0.0	7	0.0
**25677**	G	A	14040	0.0	31970	0.0	56	0.0	81	0.0	12091	13.8	13	0.0	3	0.0	9	0.0
**25785**	G	A	9363	0.0	31450	0.0	27	0.0	53	0.0	9595	14.9	6	0.0	1	0.0	6	0.0
**25789**	T	C	11930	0.0	32893	0.0	37	0.0	71	0.0	10905	6.3	12	0.0	1	0.0	8	0.0
**25892**	T	C	5982	0.0	14331	0.0	15	0.0	99	0.0	4814	5.7	3	0.0	424	0.0	5	0.0
**25934**	A	G	12216	0.0	39205	0.0	997	0.0	284	0.0	4682	5.8	6	0.0	1559	0.0	102	0.0
**26019**	A	G	14574	0.0	40144	0.0	1174	0.0	336	0.0	4907	5.5	6	0.0	1848	0.0	145	0.0
**26788**	G	A	2984	0.0	17121	0.0	4	0.0	9	0.0	999	10.7	8	0.0	0	0.0	6	0.0
**26895**	C	T	10907	0.0	27539	0.0	29	24.1	277	0.0	8913	32.6	9	0.0	0	0.0	20	0.0
**27014**	G	A	10617	0.0	27763	0.0	28	25.0	262	0.0	8755	0.0	9	0.0	0	0.0	18	0.0
**27085**	C	T	9397	0.0	23364	0.0	65	0.0	2096	0.0	26165	0.0	18	0.0	2	100.0	20	0.0
**27494**	C	T	1839	0.0	14224	6.7	3	0.0	43	0.0	1253	0.0	4	0.0	6	0.0	5	0.0
**27614**	T	C	13140	0.0	20306	0.0	13	0.0	29	0.0	4163	9.6	7	0.0	413	0.0	6	0.0
**27652**	T	C	13381	0.0	25860	0.0	47	0.0	29	0.0	9013	0.0	6	0.0	1068	45.6	7	0.0
**28494**	T	C	1854	0.0	10932	0.0	145	11.0	24	0.0	1	0.0	3	0.0	154	0.0	2	0.0
**28544**	A	G	21304	0.0	35266	0.0	170	0.0	67	0.0	2874	6.9	9	0.0	170	0.0	15	0.0
**28775**	C	T	7224	0.0	31149	0.0	4	0.0	144	0.0	95	0.0	8	0.0	807	45.0	7	0.0
**28881**	G	A	8982	0.0	15939	0.0	150	3.3	35	100.0	2569	100.0	17	100.0	1	100.0	6	83.3
**28882**	G	A	15071	0.0	22435	0.0	209	2.9	24	100.0	2110	100.0	10	100.0	0	0.0	4	75.0
**28883**	G	C	15084	0.0	22428	0.0	208	2.4	34	100.0	2533	100.0	15	100.0	1	100.0	6	83.3
**28916**	G	A	13707	0.0	27841	0.0	201	0.0	175	7.4	3988	0.0	34	0.0	105	0.0	88	0.0
**28933**	T	C	11432	0.8	27048	0.4	175	0.0	140	0.0	3486	0.0	27	0.0	99	56.6	80	0.0
**29061**	C	T	8528	0.0	21694	0.0	8	0.0	5	0.0	266	10.5	95	0.0	12	0.0	7	0.0
**29314**	A	G	5754	0.0	25412	0.2	117	0.0	23	0.0	924	0.0	8	0.0	107	7.5	9	0.0
**29555**	C	T	2937	100.0	17539	100.0	6	100.0	11	0.0	0	0.0	12	0.0	0	0.0	7	0.0
**29609**	T	C	6513	0.0	32394	0.0	213	0.0	141	0.0	7676	5.0	14	0.0	203	0.0	9	0.0
**29775**	T	C	6349	0.0	25897	0.0	242	0.0	394	0.0	10204	2.5	133	0.0	703	51.2	298	0.0

Supplementary Table S5. List of the significantly associated variants used for PRS calculation based on the A2_ALL dataset of "very severe respiratory confirmed covid" (n=2,972) vs. population (n=284,472).

CHR	POS	REF	ALT	rsid	Meta Effect Size	Meta P-value	Meta AF	Portuguese population Cohort AF
**1**	26504654	G	A	rs185041400	4.8613	3.55E-06	0.001845	0.00196
**1**	44377503	G	A	rs4314918	-0.19251	1.64E-06	0.7941	0.76932
**1**	112764378	C	T	rs2919285	-0.19459	2.15E-07	0.8032	0.78099
**1**	112785942	C	G	rs7538140	0.16488	9.57E-06	0.2219	0.21499
**1**	203954605	T	C	rs188564545	2.4201	4.13E-06	0.00984	0.00158
**1**	231996139	A	C	rs75711735	0.58372	5.52E-06	0.0374	0.02181
**2**	43251821	G	A	rs56186825	1.6652	3.27E-06	0.008928	0.00517
**2**	130431716	C	T	rs10210034	-0.14541	5.68E-06	0.5131	0.48767
**2**	137341234	A	T	rs531390248	3.36	7.77E-06	0.002369	0.00016
**2**	217725954	C	T	rs6722107	0.90977	7.93E-06	0.008955	0.01814
**3**	38579153	A	G	rs112661205	2.2515	8.46E-06	0.003901	0.00245
**3**	39204989	C	T	rs148889878	1.3637	1.70E-06	0.00486	0.00941
**3**	45889921	A	T	rs35081325	0.70561	3.82E-39	0.09694	0.05894
**3**	45908859	G	A	rs75826707	0.71416	4.54E-13	0.04609	0.0089
**3**	46032388	G	C	NA	-0.18152	9.03E-08	0.6364	0.68145
**3**	46049765	T	C	rs13433997	0.42141	3.66E-20	0.1564	0.10061
**3**	46119791	T	C	rs13434336	0.20352	1.81E-09	0.3334	0.39426
**3**	46222037	A	G	rs115102354	0.50009	3.25E-13	0.07706	0.02963
**3**	46227171	T	G	rs13062450	0.28632	6.78E-08	0.1518	0.08022
**3**	46306474	T	C	rs7631853	0.34086	2.20E-09	0.1289	0.06356
**3**	69056121	G	A	rs76821671	0.24803	5.41E-06	0.08798	0.07825
**3**	112914296	A	G	rs182721950	1.8166	6.32E-06	0.001878	0.01382
**3**	143251664	A	G	rs62269771	0.33933	7.46E-06	0.08874	0.1556
**3**	145864511	G	A	rs965032	-0.16202	2.04E-06	0.6111	0.65497
**3**	159270026	G	C	rs113427422	0.48165	2.85E-06	0.0559	0.0693
**3**	165154092	C	A	NA	-0.14427	7.20E-06	0.4587	0.40861
**4**	25475602	A	G	rs4697099	-0.23761	8.97E-06	0.1697	0.12552
**4**	36530269	C	G	rs61796478	1.5601	2.62E-06	0.008746	0.01632
**4**	122910410	G	A	rs7686809	-0.50162	1.74E-07	0.9453	0.88729
**5**	7505930	C	T	rs192229354	3.5975	4.20E-06	0.002109	0.00484
**5**	17130850	T	C	rs790210	0.54784	5.30E-06	0.03515	5.00E-05
**5**	17142133	G	T	rs645922	0.58518	4.78E-06	0.03495	0.02149
**5**	24843084	A	G	rs141962254	2.9487	6.74E-06	0.001148	0.0015
**5**	58786782	G	T	rs146410305	0.67245	2.24E-06	0.02408	0.0062
**5**	79070021	C	T	rs114128029	1.1891	1.60E-06	0.01538	0.00427
**5**	131740656	A	C	rs13168774	-0.19091	6.61E-06	0.8634	0.83698
**5**	154604690	C	G	NA	0.2305	8.22E-06	0.1513	0.07799
**5**	164850943	G	A	rs6880269	-0.2311	4.99E-06	0.5075	0.50703
**6**	7549628	C	T	rs2299036	0.29664	9.19E-07	0.1795	0.17953
**6**	31121426	G	A	rs143334143	0.45345	5.60E-17	0.1437	0.12398
**6**	32667171	A	T	rs1794280	-0.30549	1.71E-08	0.1002	0.09167
**6**	33055355	A	G	NA	0.27651	2.76E-07	0.07587	0.09132
**6**	41719110	T	C	NA	-0.56103	7.10E-06	0.976	0.96753
**6**	88576981	T	C	rs6935448	-0.22002	5.42E-06	0.8219	0.83752
**6**	98715160	G	A	rs117937941	0.47232	2.25E-06	0.005898	0.04364
**7**	37831003	A	G	rs183729083	1.8366	9.03E-06	0.005809	0.00277
**7**	54647894	A	C	rs622568	0.27338	2.83E-10	0.1608	0.07366
**7**	88639135	T	C	rs78211246	-0.19717	6.19E-06	0.1759	0.17091
**7**	107607902	C	T	rs2237698	0.22516	6.99E-06	0.09765	0.1192
**7**	113317708	T	C	rs12705891	0.16763	6.31E-07	0.3918	0.41102
**8**	1779298	C	T	rs180717749	0.69669	4.98E-06	0.002375	0.00724
**8**	71700365	C	T	rs147667474	2.0373	2.89E-06	0.01164	0.00431
**8**	141857912	A	G	rs149938155	0.43195	5.95E-06	0.01057	0.0238
**9**	123550027	G	A	NA	0.28206	9.25E-06	0.7073	0.7653
**10**	33178246	A	C	NA	-0.21564	6.72E-06	0.8609	0.86629
**10**	44340072	T	A	rs118052809	0.55026	2.60E-06	0.01048	0.01291
**10**	97252761	A	T	rs117098321	1.0297	3.69E-07	0.008664	0.01872
**11**	22828273	C	T	rs78594643	-0.54047	9.33E-06	0.01995	0.02303
**11**	35402078	T	C	rs1923302	-0.17211	9.34E-06	0.2239	0.21984
**11**	44727667	A	G	rs4450162	0.46666	3.97E-06	0.04025	0.06024
**11**	125792416	T	C	rs662722	-0.24615	5.55E-06	0.7416	0.67452

CHR	POS	REF	ALT	rsid	Meta Effect Size	Meta P-value	Meta AF	Portuguese population Cohort AF
**12**	29229856	G	A	NA	-0.19661	2.75E-06	0.8177	0.81272
**12**	103014757	C	A	NA	-0.37565	1.98E-14	0.8746	0.86556
**12**	113381956	C	T	rs2269899	0.21454	8.55E-10	0.72	0.62316
**12**	113385375	T	C	rs10850104	0.19459	2.36E-07	0.3694	0.19024
**13**	44342409	A	G	rs9533610	-0.20652	7.76E-06	0.8439	0.81248
**13**	44604709	C	G	NA	0.47437	2.01E-06	0.007279	0.02136
**13**	67940439	A	T	rs9592514	2.5513	5.71E-06	0.00228	0.00378
**14**	81096699	C	T	rs8016670	-0.27455	3.96E-06	0.9602	0.91986
**15**	37079890	A	G	rs149402468	1.9556	6.47E-06	0.003407	0.0086
**15**	79766794	G	A	NA	-0.1936	1.42E-06	0.2287	0.23173
**15**	99973286	T	G	rs74035732	-0.35901	7.65E-06	0.04311	0.05008
**17**	10101496	C	T	rs149399480	1.8031	8.46E-07	0.006129	0.00804
**17**	17462512	C	T	rs568997551	2.2931	3.45E-06	0.01207	0.00343
**17**	33976296	C	T	NA	-0.18834	7.91E-06	0.7955	0.80944
**17**	76252183	G	A	NA	0.36136	2.62E-06	0.1979	0.14406
**18**	36300243	A	G	rs142354687	3.8192	5.03E-06	0.002618	0.00375
**19**	4723670	C	A	NA	0.25516	6.69E-13	0.3364	0.27724
**19**	10427721	T	A	NA	0.38141	1.31E-07	0.03602	0.03906
**19**	10466123	C	T	rs11085727	0.18858	1.23E-07	0.267	0.30801
**19**	10596988	C	A	rs45524632	0.51705	8.67E-07	0.01571	0.01407
**19**	17505834	G	C	NA	0.30266	3.32E-06	0.7688	0.82365
**19**	43266536	C	G	rs112599803	0.66574	2.48E-06	0.06424	0.04464
**19**	43905258	C	T	rs75028208	1.3477	2.23E-06	0.01459	0.03103
**19**	56456647	G	A	rs141789023	0.56968	2.89E-06	0.1156	0.03703
**20**	24479907	C	T	rs547278572	2.8651	5.12E-06	0.002402	0.00149
**20**	32781699	G	C	rs546079703	2.4138	1.72E-07	0.004147	0.00014
**20**	37563008	T	C	rs186431128	2.7074	5.59E-06	0.002141	0.00028
**20**	57549023	C	T	rs150240336	1.4451	5.30E-06	0.0143	0.01093
**21**	34615210	T	C	rs13050728	-0.19739	1.84E-08	0.6114	0.69188
**22**	41252291	T	C	rs192261735	0.63286	3.12E-06	0.006277	0.0072

Supplementary Table S6. List of the significantly associated variants used for PRS calculation based on the B2_ALL dataset of "hospitalised covid" (n=6,492) vs. population (n=1,012,809).

CHR	POS	REF	ALT	rsid	Meta Effect Size	Meta P-value	Meta AF	Portuguese population Cohort AF
**1**	53771860	G	A	rs115038483	-0.42964	6.43E-06	0.01405	0.01063
**1**	65449821	G	A	rs4454580	0.18513	2.22E-06	0.1157	0.13099
**1**	91208514	A	C	rs2166172	0.10713	8.63E-06	0.4079	0.35832
**1**	237277098	A	C	rs9287218	-0.24463	2.82E-06	0.05047	0.04674
**2**	162936216	C	T	rs117888248	0.6907	2.37E-06	0.01038	0.02342
**2**	182809457	C	G	rs74799459	0.30726	9.18E-06	0.03178	0.04498
**2**	195035942	A	G	rs62186769	0.61202	8.87E-06	0.008733	0.00035
**3**	7794348	C	T	NA	-0.14468	9.44E-06	0.2636	0.24427
**3**	27526516	C	T	rs6771541	0.14359	1.17E-06	0.3799	0.44167
**3**	45553090	G	A	rs79939301	0.25641	5.57E-07	0.08346	0.02906
**3**	45798226	C	T	rs17213127	0.38764	7.66E-08	0.04395	0.0246
**3**	45818880	G	C	NA	0.21697	3.02E-06	0.1237	0.11258
**3**	45822010	T	C	rs73062378	0.18408	8.04E-06	0.2093	0.15026
**3**	45889921	A	T	rs35081325	0.59959	9.52E-50	0.08054	0.05894
**3**	45908859	G	A	rs75826707	0.64829	3.59E-16	0.03148	0.0089
**3**	45910870	G	A	rs2191031	0.21	2.31E-09	0.2034	0.18051
**3**	46042413	A	T	NA	-0.15672	7.31E-11	0.5993	0.66127
**3**	46047767	G	C	rs4234452	-0.10771	7.36E-06	0.4014	0.38653
**3**	46049765	T	C	rs13433997	0.37339	1.66E-29	0.1322	0.10061
**3**	46093858	G	A	rs13098271	0.15548	2.01E-10	0.3283	0.37582
**3**	46194589	C	A	rs71327036	0.28439	8.85E-14	0.1052	0.06948
**3**	46222037	A	G	rs115102354	0.45004	1.97E-18	0.06527	0.02963
**3**	46301423	T	A	rs11919884	0.27677	5.95E-12	0.09721	0.07677
**3**	46464017	A	C	rs34671664	0.19128	3.12E-07	0.1241	0.06349
**4**	36530269	C	G	rs61796478	1.0631	1.66E-06	0.005496	0.01632

CHR	POS	REF	ALT	rsid	Meta Effect Size	Meta P-value	Meta AF	Portuguese population Cohort AF
**4**	142430667	A	T	NA	0.4692	1.54E-06	0.02485	0.02312
**5**	56485892	G	A	rs55737726	0.59065	3.43E-06	0.01254	0.00438
**5**	58786782	G	T	rs146410305	0.54762	1.08E-06	0.01726	0.0062
**5**	65066483	C	T	rs114969787	0.26622	6.51E-06	0.03846	0.06299
**5**	71698763	T	C	rs187920931	1.857	1.43E-06	0.004279	0.00035
**5**	71763420	A	G	rs139478596	2.0038	5.83E-07	0.004027	5.10E-04
**5**	71893271	G	A	rs191971874	2.0461	7.90E-07	0.003874	0.00239
**5**	131740656	A	C	rs13168774	-0.14334	2.62E-06	0.8344	0.83698
**5**	162727453	C	T	rs79833209	0.37801	2.64E-06	0.02819	0.01345
**6**	31121426	G	A	rs143334143	0.2646	3.45E-11	0.08689	0.12398
**6**	33055355	A	G	NA	0.18117	3.58E-06	0.08416	0.09132
**6**	41497035	C	A	NA	0.26593	8.86E-10	0.1251	0.09466
**6**	41501834	G	A	rs12175265	0.43782	4.55E-09	0.03502	0.01744
**6**	108279068	G	A	rs200955319	1.0422	6.61E-07	0.006912	0.00534
**6**	157867030	C	T	rs9364501	-0.12713	6.96E-06	0.4899	0.45094
**7**	53493705	G	A	rs12718791	-0.17468	3.32E-06	0.7783	0.84959
**7**	54647894	A	C	rs622568	0.16634	2.77E-07	0.1477	0.07366
**7**	107607902	C	T	rs2237698	0.16851	2.69E-06	0.09467	0.1192
**8**	9875204	T	C	rs1396186	0.16268	2.64E-06	0.2264	0.20498
**8**	10010765	G	C	NA	0.15341	7.41E-06	0.1962	0.18969
**8**	15343283	T	C	rs114717629	1.1699	9.23E-06	0.00655	0.00713
**8**	59903271	T	C	rs7823862	-0.20454	1.65E-06	0.06634	0.08014
**8**	110059637	A	C	rs189378134	1.0541	7.28E-07	0.007647	0.0043
**8**	110339840	T	A	rs144837205	0.96534	3.58E-06	0.007712	0.00424
**9**	15397969	A	C	rs2798716	-0.18197	6.51E-06	0.9302	0.87977
**9**	26974745	C	T	rs150788916	1.4433	3.49E-06	0.004252	0.00346
**10**	12667474	C	G	rs4310517	-0.1396	6.65E-06	0.7521	0.6526
**10**	20642449	T	C	NA	1.5167	6.14E-06	0.004527	0.00035
**10**	44340072	T	A	rs118052809	0.45242	1.31E-06	0.02222	0.01291
**10**	70172332	G	A	NA	0.35143	5.77E-06	0.02673	0.02881
**10**	121010105	A	C	rs112969140	0.20563	8.00E-06	0.08061	0.05773
**11**	1005506	A	G	rs147685259	1.2734	4.82E-06	0.006075	0.00526
**11**	113106528	G	T	NA	-0.27335	2.90E-06	0.05598	0.04889
**12**	31392117	C	T	rs75594480	0.90255	4.16E-06	0.01692	0.01057
**12**	103014757	C	A	NA	-0.18231	5.17E-07	0.886	0.86556
**12**	113362997	T	G	NA	0.14763	5.74E-09	0.6768	0.61171
**12**	129582079	T	C	rs116993182	0.33965	1.80E-06	0.01796	0.02046
**13**	73889735	T	C	rs2325521	0.28856	9.99E-06	0.03812	0.01865
**14**	38848003	A	G	rs1754680	-0.11565	1.90E-06	0.3974	0.39369
**15**	26780764	T	G	NA	0.49562	5.17E-06	0.04097	0.01152
**15**	55197554	G	T	NA	1.1663	9.95E-06	0.009457	0.00387
**15**	95579943	A	G	rs145572293	0.61208	2.99E-06	0.01468	0.01191
**17**	2846004	G	A	rs55818593	0.18055	1.50E-06	0.3504	0.33101
**17**	61954669	G	A	rs145032579	0.65208	6.12E-06	0.009317	0.00884
**18**	25179662	A	G	rs146116110	0.69167	3.65E-06	0.01124	0.00729
**19**	4723670	C	A	NA	0.18122	2.31E-12	0.315	0.27724
**19**	10423815	G	A	rs8101195	-0.13932	7.99E-06	0.8307	0.80739
**19**	10427721	T	A	NA	0.32939	4.50E-09	0.0506	0.03906
**19**	10596988	C	A	rs45524632	0.47598	4.62E-09	0.02362	0.01407
**21**	25507072	G	C	rs190545393	1.4221	4.45E-06	0.004305	0.00712
**21**	34610487	T	C	rs1131964	0.13008	5.50E-08	0.5761	0.51179
**21**	34615210	T	C	rs13050728	-0.17812	8.83E-13	0.6515	0.69188
**21**	34620801	A	G	rs2073362	0.2271	3.81E-08	0.08026	0.07966
**22**	19568533	C	T	rs9604980	0.18951	2.19E-07	0.1053	0.07951

Supplementary Table S7. PRS score values for the “very severe respiratory confirmed covid” and “hospitalised covid” phenotypes in the patient and individuals from the Portuguese cohort.

ID	“very severe respiratory confirmed covid”	“hospitalised covid”
**Patient**	-4.87655	-0.03313
**Control_1**	-4.90E+00	-1.97551
**Control_2**	-5.16E+00	-1.89236
**Control_3**	-7.15E+00	-0.54649
**Control_4**	-4.89E+00	-1.71186
**Control_5**	-6.21E+00	-0.68627
**Control_6**	-3.59E+00	-1.72808
**Control_7**	2.68E-01	1.37396
**Control_8**	-5.00E+00	-0.4512
**Control_9**	-3.28E+00	-0.24699
**Control_10**	-5.32E+00	-1.14481
**Control_11**	-4.04E+00	-1.56374
**Control_12**	-2.56E+00	-0.64326
**Control_13**	-4.67E-01	0.62717
**Control_14**	-5.08E+00	-1.36083
**Control_15**	-4.77E+00	-0.51723
**Control_16**	-5.29E+00	-0.59627
**Control_17**	-3.30E+00	-0.64868
**Control_18**	-4.98E+00	0.18003
**Control_19**	-5.35E+00	-0.09047
**Control_20**	-5.00E+00	-1.34065
**Control_21**	-3.38E+00	-0.32906
**Control_22**	-2.80E+00	-1.0804
**Control_23**	-4.50E+00	-0.59643
**Control_24**	-1.03E+00	-0.39641
**Control_25**	-4.99E+00	1.67317
**Control_26**	-4.36E+00	-0.57461
**Control_27**	-4.04E+00	0.22938
**Control_28**	-4.70E+00	-0.18751
**Control_29**	-5.64E+00	0.14805
**Control_30**	-4.89E+00	0.60239
**Control_31**	-4.69E+00	-1.2565
**Control_32**	-2.93E+00	1.90648
**Control_33**	-4.09E+00	0.69502
**Control_34**	-6.03E+00	-2.02994
**Control_35**	-9.81E-01	-1.1441
**Control_36**	-3.89E+00	-0.97245
**Control_37**	-4.96E+00	-0.34381
**Control_38**	-5.23E+00	-0.97005
**Control_39**	-5.39E+00	0.94992
**Control_40**	-4.90E+00	-1.05645
**Control_41**	-3.18E+00	0.21109
**Control_42**	-5.11E-01	0.85563
**Control_43**	-4.98E+00	-1.45702
**Control_44**	-4.27E+00	0.3355
**Control_45**	-6.39E+00	-1.62096
**Control_46**	-3.61E+00	-1.34278
**Control_47**	-7.10E+00	-0.84061
**Control_48**	-4.59E+00	-0.6492
**Control_49**	-4.75E+00	-2.19425
**Control_50**	-2.00E+00	0.68209
**Control_51**	-2.90E+00	0.75568
**Control_52**	-4.35E+00	-0.85561
**Control_53**	-4.19E+00	0.33753
**Control_54**	-3.04E+00	-0.08448
**Control_55**	-5.70E+00	1.603
**Control_56**	-6.32E+00	-1.5768
**Control_57**	-6.29E+00	-0.7849
**Control_58**	-5.36E+00	0.25457
**Control_59**	-5.56E+00	-1.21327
**Control_60**	-3.79E+00	-0.07656
**Control_61**	-5.41E+00	-0.10805
**Control_62**	-4.54E+00	-0.31598

ID	“very severe respiratory confirmed covid”	“hospitalised covid”
**Control_63**	-3.18E+00	1.7555
**Control_64**	-4.35E+00	-0.67707
**Control_65**	-3.89E+00	0.66665
**Control_66**	-4.08E+00	-0.62997
**Control_67**	-3.23E+00	-0.17569
**Control_68**	-4.96E+00	-0.05921
**Control_69**	-4.50E+00	0.30167
**Control_70**	-4.09E+00	-0.61154
**Control_71**	-5.43E+00	-1.2121
**Control_72**	-1.56E+00	3.75514
**Control_73**	-4.86E+00	-1.09983
**Control_74**	-4.61E+00	0.20464
**Control_75**	-4.76E+00	-1.63796
**Control_76**	-3.79E+00	0.8922
**Control_77**	-2.17E+00	3.44269
**Control_78**	-4.49E+00	-0.05917
**Control_79**	-4.28E+00	-0.52001
**Control_80**	-3.55E+00	-1.62611
**Control_81**	-4.43E+00	-1.15316
**Control_82**	-5.01E+00	0.56405
**Control_83**	-3.23E+00	-1.33503
**Control_84**	-1.77E+00	2.4039
**Control_85**	-5.38E+00	-0.09866
**Control_86**	-2.50E+00	0.87392
**Control_87**	-2.11E+00	-0.56459
**Control_88**	-1.73E+00	0.06622
**Control_89**	-2.95E+00	0.2116
**Control_90**	-6.24E+00	-1.60651
**Control_91**	-2.30E+00	-1.53324
**Control_92**	-6.99933	-1.61907
**Control_93**	-2.45405	1.48303
**Control_94**	-4.34987	1.67316
**Control_95**	-4.72626	0.38721
**Control_96**	-4.80856	-0.0857
**Control_97**	-3.04659	0.82665
**Control_98**	-2.38563	0.20145
**Control_99**	-2.43129	-1.40496
**Control_100**	-5.86669	0.14784
**Control_101**	-1.35701	0.68653
**Control_102**	-1.23382	-1.78844
**Control_103**	-4.10605	-0.09449
**Control_104**	-0.39854	1.65477
**Control_105**	-3.63653	0.12011
**Control_106**	-4.61694	-0.25435
**Control_107**	-4.68681	-0.63197
**Control_108**	-3.23328	-0.08327
**Control_109**	-2.97989	0.92944
**Control_110**	-4.43776	-0.95831
**Control_111**	-2.198	1.2198
**Control_112**	2.42573	3.37488
**Control_113**	-4.404	-0.21011
**Control_114**	-2.81878	0.12863
**Control_115**	-6.21434	0.26661
**Control_116**	-3.78205	-1.04036
**Control_117**	-4.66823	-0.25966
**Control_118**	-4.38954	-0.68852
**Control_119**	0.30253	0.48275
**Control_120**	-6.90608	-1.78801
**Control_121**	-4.67019	-1.55847
**Control_122**	-5.11919	1.00139
**Control_123**	-5.31657	-0.20587
**Control_124**	-2.34435	-0.87753
**Control_125**	-3.97191	0.56356
**Control_126**	-5.72312	-1.35796
**Control_127**	-4.12922	-0.98348
**Control_128**	-1.64077	-0.44908
**Control_129**	-5.41002	-1.11184
**Control_130**	-0.49236	-1.32733
**Control_131**	-4.5782	-0.15519

ID	"very severe respiratory confirmed covid"	"hospitalised covid"
**Control_132**	-5.70871	0.34212
**Control_133**	-4.67043	-0.68724
**Control_134**	-3.25457	-0.45825
**Control_135**	-6.27244	-2.13391
**Control_136**	-3.55693	-0.02005
**Control_137**	-2.1284	-1.45675
**Control_138**	-3.72811	-0.22451
**Control_139**	-0.47755	-0.6321
**Control_140**	-4.73245	0.13417
**Control_141**	-5.72684	-0.78091
**Control_142**	-3.63986	-0.04767
**Control_143**	-3.32291	-1.64979
**Control_144**	-3.42449	-0.85886
**Control_145**	-4.63228	0.18671
**Control_146**	-4.94364	-0.26755
**Control_147**	-6.41413	-1.58766
**Control_148**	-3.53471	-0.61872
**Control_149**	-5.57549	-1.11323
**Control_150**	-4.74915	-0.87604
**Control_151**	-4.09005	0.18534
**Control_152**	-5.56207	-1.43691
**Control_153**	-4.68608	-1.04662
**Control_154**	-3.91342	0.93384
**Control_155**	-7.52479	-2.15656
**Control_156**	-4.82207	-1.29347
**Control_157**	-3.18609	-1.0193
**Control_158**	-4.86976	-2.02661
**Control_159**	-2.87761	-0.26275
**Control_160**	-4.93477	-0.12399
**Control_161**	-3.21235	1.42861
**Control_162**	-2.77394	0.4151
**Control_163**	-1.9014	0.94125
**Control_164**	-1.97826	0.78863
**Control_165**	-4.09434	-0.14756
**Control_166**	-4.74327	-1.01587
**Control_167**	-4.39397	-0.76348
**Control_168**	-4.45865	-0.8555
**Control_169**	-3.41697	1.12599
**Control_170**	-2.99592	1.23925
**Control_171**	-4.68866	0.27114
**Control_172**	-5.49796	-0.28772
**Control_173**	-4.87907	1.22099
**Control_174**	-4.09014	-1.58057
**Control_175**	-5.75143	-0.33986
**Control_176**	-3.40585	0.07098
**Control_177**	-5.17761	-0.7763
**Control_178**	-4.0947	-0.68351
**Control_179**	-5.09591	0.17281
**Control_180**	-4.41746	1.55421
**Control_181**	-6.23705	-0.33483
**Control_182**	-5.67844	-0.31867
**Control_183**	-4.6586	-0.42976
**Control_184**	-5.43925	-0.65352
**Control_185**	-3.61809	0.00716
**Control_186**	-5.14192	0.20938
**Control_187**	-3.33742	-0.53662
**Control_188**	-4.92869	0.53324
**Control_189**	-3.41719	0.165
**Control_190**	-3.15799	-0.58069
**Control_191**	-4.90514	-0.88877
**Control_192**	-4.80698	-1.24858
**Control_193**	-4.67025	1.04502
**Control_194**	-1.87099	0.61465
**Control_195**	-4.43074	0.39739
**Control_196**	-3.47731	0.58579
**Control_197**	-4.36153	0.31097
**Control_198**	-5.80403	-0.2157
